# First systematic experimental 2D mapping of linearly polarized *γ*-ray polarimetric distributions in relativistic Compton scattering

**DOI:** 10.1093/nsr/nwag073

**Published:** 2026-02-09

**Authors:** Kaijie Chen, Xiangfei Wang, Hanghua Xu, Gongtao Fan, Zhenwei Wang, Zirui Hao, Longxiang Liu, Yue Zhang, Sheng Jin, Zhicai Li, Pu Jiao, Qiankun Sun, Mengdie Zhou, Yulong Shen, Mengke Xu, Chang Yang, Jiawen Ding, Hongwei Wang, Wenqing Shen, Yugang Ma

**Affiliations:** School of Physical Science and Technology, ShanghaiTech University, Shanghai 201210, China; Shanghai Institute of Applied Physics, Chinese Academy of Sciences, Shanghai 201800, China; University of Chinese Academy of Sciences, Beijing 101408, China; Shanghai Advanced Research Institute, Chinese Academy of Sciences, Shanghai 201210, China; Shanghai Institute of Applied Physics, Chinese Academy of Sciences, Shanghai 201800, China; University of Chinese Academy of Sciences, Beijing 101408, China; Shanghai Advanced Research Institute, Chinese Academy of Sciences, Shanghai 201210, China; Shanghai Institute of Applied Physics, Chinese Academy of Sciences, Shanghai 201800, China; University of Chinese Academy of Sciences, Beijing 101408, China; Shanghai Institute of Applied Physics, Chinese Academy of Sciences, Shanghai 201800, China; University of Chinese Academy of Sciences, Beijing 101408, China; Shanghai Advanced Research Institute, Chinese Academy of Sciences, Shanghai 201210, China; Shanghai Advanced Research Institute, Chinese Academy of Sciences, Shanghai 201210, China; Shanghai Advanced Research Institute, Chinese Academy of Sciences, Shanghai 201210, China; Shanghai Institute of Applied Physics, Chinese Academy of Sciences, Shanghai 201800, China; University of Chinese Academy of Sciences, Beijing 101408, China; Shanghai Advanced Research Institute, Chinese Academy of Sciences, Shanghai 201210, China; Shanghai Advanced Research Institute, Chinese Academy of Sciences, Shanghai 201210, China; Shanghai Institute of Applied Physics, Chinese Academy of Sciences, Shanghai 201800, China; University of Chinese Academy of Sciences, Beijing 101408, China; Shanghai Advanced Research Institute, Chinese Academy of Sciences, Shanghai 201210, China; Shanghai Advanced Research Institute, Chinese Academy of Sciences, Shanghai 201210, China; Shanghai Institute of Applied Physics, Chinese Academy of Sciences, Shanghai 201800, China; University of Chinese Academy of Sciences, Beijing 101408, China; Shanghai Advanced Research Institute, Chinese Academy of Sciences, Shanghai 201210, China; Shanghai Institute of Applied Physics, Chinese Academy of Sciences, Shanghai 201800, China; University of Chinese Academy of Sciences, Beijing 101408, China; Shanghai Advanced Research Institute, Chinese Academy of Sciences, Shanghai 201210, China; Shanghai Institute of Applied Physics, Chinese Academy of Sciences, Shanghai 201800, China; University of Chinese Academy of Sciences, Beijing 101408, China; Shanghai Advanced Research Institute, Chinese Academy of Sciences, Shanghai 201210, China; University of Chinese Academy of Sciences, Beijing 101408, China; School of Physical Science and Technology, ShanghaiTech University, Shanghai 201210, China; Key Laboratory of Nuclear Physics and Ion-beam Application, and Institute of Modern Physics, Fudan University, Shanghai 200433, China; School of Physics, East China Normal University, Shanghai 200062, China

**Keywords:** inverse Compton scattering, Shanghai laser electron gamma source, degree of polarization, angle of polarization

## Abstract

The interaction of photons with relativistic electrons constitutes a fundamental electromagnetic process whose polarization-transfer mechanics remain incompletely characterized. We report the first systematic measurement of the spatial polarization distribution for $\gamma$ rays generated via $45 {}^{\circ }$ slant inverse Compton scattering (ICS) between linearly polarized $0.117 \,\mathrm{eV}$ photons and $3.5 \,\mathrm{GeV}$ electrons, performing full two-dimensional mapping of the intensity, angle of polarization (AOP) and degree of polarization (DOP). The measurements reveal an asymmetric beam profile along the laser polarization direction that resembles observations from $180 {}^{\circ }$ backward ICS. The central beam region exhibits DOP near 1.0, with the AOP rigidly aligned at $45 {}^{\circ }$, while peripheral regions display complex, non-uniform polarization distributions. These findings confirm quantum electrodynamics predictions of near-complete polarization transfer along the beam axis in slant geometries, thereby establishing slant scattering as a viable alternative to head-on configurations for generating high-DOP $\gamma$ rays.

## Teaser

This work presents the first systematic 2D mapping of intensity and polarization (AOP/DOP) of ? rays from 45° slant inverse Compton scattering, demonstrating near-complete on-axis polarization transfer and establishing slant geometry as a viable source of highly polarized ? radiation.

## INTRODUCTION

The interaction of photons with electrons is one of the most fundamental electromagnetic processes and has been systematically studied over a long history since the 1920s [1,2]. Within the theoretical framework of quantum electrodynamics (QED), scattered photons naturally inherit the polarization information of the incident photons. If the incident electrons are relativistic—in other words, endowed a high momentum—then scattered photons propagate predominantly along the direction of the electron momentum [3–7].

The properties of photons scattered from electrons can be well described by QED [6]. Especially in the non-relativistic case, by employing the Lorentz-invariant Stokes parameters $\xi _i$ [8], the spatial distributions of the degree of polarization (DOP) and angle of polarization (AOP) can be obtained [9,10]. For instance, in interactions between completely linearly polarized incident photons ($\xi _2=0$, $\xi _1^2 + \xi _3^2=1$) and non-relativistic electrons, QED predicts that the scattered photons remain linearly polarized, with the spatial intensity distribution exhibiting azimuthal asymmetry [4,6]. Furthermore, non-relativistic Compton scattering of polarized photons by electrons has been experimentally validated with high precision and successfully applied in Compton camera technology [11] and astrophysics [12–14].

In relativistic regimes, however, QED-based descriptions of inverse Compton-scattered photon properties necessitate more sophisticated treatments, and approximations remain nearly unavoidable during theoretical derivations and calculations [3,15,16]. Recently, multiple research groups have simulated polarization distributions of photons scattered from collisions between polarized photon and relativistic electron beam using Monte Carlo methods within QED or classical electrodynamics frameworks, yielding consistent results [8,15–21].

Over the past two decades, experimental investigations into the polarization properties of inverse Compton scattering have progressed through several key milestones. Initial studies focused on the spatial intensity of polarized $\gamma$ rays in the standard $180 \,{}^{\circ }$ backscattering geometry [16,22]. A subsequent study achieved a significant milestone at HI$\gamma$S by quantifying the DOP of linear polarized $\gamma$ rays in this geometry, although the measurements were confined to the center of the $\gamma$-ray beam [23]. Tsinghua University also experimentally demonstrated the average DOP in the central region of Thomson-scattered X-rays [18]. More recently, research has expanded to non-standard $90 \,{}^{\circ }$ geometries at UVSOR-III and has employed advanced laser modes [24,25]. In particular, a systematic measurement of $\gamma$ rays with different polarization states was performed at different regions of the $\gamma$-ray spot [26]. Nevertheless, experimental data on the spatial distributions of the DOP and AOP of photons scattered from relativistic electrons remain neither systematic nor sufficient.

The scarcity of experimental data on the polarization properties of photons scattered from relativistic electrons hinders both theoretical and experimental research. In the absence of systematic data on the spatial distributions of the DOP and AOP of scattered photons, the above-mentioned theoretical models of photon–relativistic electron interactions cannot be systematical verified. In specialized experimental scenarios involving the inverse Compton scattering (ICS) source, such data are even more indispensable [27–34], as they directly determine the accuracy of polarization-dependent measurements [35,36]. Beyond fundamental science, these precious polarization parameters are also crucial for advancing frontier applications such as nuclear security interrogation [37,38], polarized positron sources [39] and astrophysical observation [14].

Here, we report the first systematic measurement of the spatial polarization distribution of the DOP and AOP of $\gamma$-ray photons (approximately 3 MeV) produced via $45 \,{}^{\circ }$ slant ICS between $0.117 \,\mathrm{eV}$ ($\lambda$ = $10.64 \,\mathrm{{\mu }m}$) linearly polarized $\mathrm{CO}_{2}$ laser photons and $3.5 \,\mathrm{GeV}$ relativistic electrons in the Shanghai Synchrotron Radiation Facility (SSRF) storage ring.

## RESULTS AND DISCUSSION

### Experimental set-up and method

**Figure 1.**
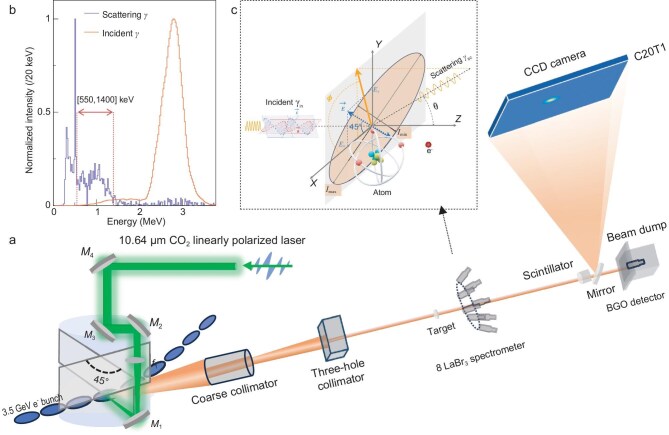
(a) Experimental set-up. The Compton scattering process between the incident $\gamma$ rays and the Ta target, together with the geometry, is illustrated in inset (c). The spectra of the incident $\gamma$ rays and the secondary scattered $\gamma$ rays from the Ta target are shown in inset (b), measured at the centre of the $\gamma$-ray beam spot.

The experiment was carried out at the Shanghai Laser Electron Gamma Source (SLEGS) beamline of the Shanghai Synchrotron Radiation Facility (SSRF) [40,41]. The experimental set-up is shown in Fig. 1.

During the experiment, the SSRF storage ring was operated in top-up mode with an electron beam current of $200 \,\mathrm{mA}$, while the $\mathrm{CO}_{2}$ laser was operated in gated continuous-wave mode using an RF-enable signal, producing pulses with a repetition frequency of $1 \,\mathrm{kHz}$ and a pulse width of $50 \,\mathrm{\mu s}$. The DOP of the incident laser was carefully measured to be $1.001\pm 0.002$, corresponding to nearly 100% polarization (see Section S1.1 of the online supplementary material). The spatial profile and intensity distribution of the $\gamma$-ray spot were recorded at a distance of 40 m downstream from the interaction point using a $\gamma$-ray imaging system [42].

The polarization state of the $\gamma$-ray beam is characterized by its DOP and AOP. The DOP quantifies the fraction of photons in a polarized state and is derived from the four Stokes parameters $\xi _{i}$ as


(1) \begin{eqnarray*}
\mathrm{DOP}=\sqrt{{\lambda \xi _1\rangle }^2+{\lambda \xi _2\rangle }^2+{\lambda \xi _3\rangle }^2},
\end{eqnarray*}

where $\lambda \xi _i \rangle$ denotes the average Stokes parameter of all photons within the collimating aperture [7]. The AOP indicates the azimuthal angle of the primary oscillation direction of the electric field vector and is calculated as $\mathrm{AOP}=(1/2)\arctan (\lambda \xi _1\rangle /\lambda \xi _3\rangle )$ [7]. To measure the transverse distributions of the AOP and DOP of the generated $\gamma$-ray beam, a $\gamma$-ray polarimeter based on Compton scattering—referred to as linearly polarized $\gamma$-ray scattering with a tantalum (Ta) target—was developed at SLEGS (see Section S2). Similar methods have been widely adopted and validated in Compton camera applications [11] and Compton polarimeter [43–47]. The geometry of Compton scattering between linearly polarized $\gamma$ rays and electrons in the Ta target in the laboratory frame is shown in Fig. 1c. In the electron rest frame, the differential cross section for Compton scattering of linearly polarized $\gamma$ rays is described by the Klein–Nishina formula:


(2) \begin{eqnarray*}
\frac{\mathrm{d}\sigma }{\mathrm{d}\Omega }(\theta ,\phi ,E_{\mathrm{in}})\! =\!\frac{r_{e}^2}{2} \bigg (\frac{E_{\mathrm{sc}}}{E_{\mathrm{in}}}\bigg )^2 \bigg (\frac{E_{\mathrm{sc}}}{E_{\mathrm{in}}} \!+\! \frac{E_{\mathrm{in}}}{E_{\mathrm{sc}}}{-}\sin ^2\Theta \bigg ),
\end{eqnarray*}

where $r_{e}$ is the classical electron radius, $E_{\mathrm{in}}$ and $E_{\mathrm{sc}}$ are the energies of the incident and scattered photons, respectively, and $\Theta$ is the angle between the two polarization vectors. In terms of the polar scattering angle $\theta$ and the azimuthal angle $\phi$ between the polarization vector of the incident $\gamma$ ray and the Compton scattering plane (see Fig. 1c), Equation (2) can be rewritten as


(3) \begin{eqnarray*}
&&\frac{\mathrm{d}\sigma }{\mathrm{d}\Omega }(\theta ,\phi ,E_{\mathrm{in}}) = \frac{r_{e}^2}{2} \bigg ( \frac{E_{\mathrm{sc}}}{E_{\mathrm{in}}} \bigg )^2 \bigg ( \frac{E_{\mathrm{sc}}}{E_{\mathrm{in}}} \bigg ) \\
&&\quad + \frac{r_{e}^2}{2} \bigg ( \frac{E_{\mathrm{sc}}}{E_{\mathrm{in}}} \bigg )^2 \bigg ( \frac{E_{\mathrm{in}}}{E_{\mathrm{sc}}} \bigg ) - \displaystyle\frac{r_{e}^2}{2} \bigg ( \frac{E_{\mathrm{sc}}}{E_{\mathrm{in}}} \bigg )^2 \\
&&\qquad\quad \times \, ( 2\sin ^2\theta \cos ^2\phi ).
\end{eqnarray*}

The energies $E_{\mathrm{in}}$ and $E_{\mathrm{sc}}$ are related by


(4) \begin{eqnarray*}
E_{\mathrm{sc}}=\frac{E_{\mathrm{in}}}{1 + \frac{E_{\mathrm{in}}}{m_{e}c^2} (1-\cos \theta )} .
\end{eqnarray*}

Equation (3) shows that the azimuthal distribution of secondary Compton photons is of a $\mathrm{cos^2\phi }$-like shape. In this experiment, a Ta target at room temperature ($T=300 \,\mathrm{K}$) was used. The root-mean-square speed of electrons in the target, which follow the Maxwell–Boltzmann distribution, is $v_\mathrm{rms}=\sqrt{3kT/m}=1.17 \times 10^{5} \,{\rm m}/{\rm s}$, which is much smaller than the speed of light ($v_\mathrm{rms}/c=3.8 \times 10^{-4}$, corresponding to a Lorentz factor $\gamma _{L} \approx 1$). Therefore, Equations (2) and (3) in the laboratory frame can be approximated as identical to their forms in the electron rest frame. The differential cross section reaches its extreme values at $\phi =0 {}^{\circ }$ and $90 {}^{\circ }$. Their ratio, an observable quantity, is referred to as the peak-to-valley ratio (PVR):


(5) \begin{eqnarray*}
\mathrm{PVR} = \frac{{\frac{\mathrm{d}\sigma } {\mathrm{d}\Omega} }(\theta ,90 {}^{\circ },E_{\mathrm{in}})} {\frac{\mathrm{d}\sigma}{\mathrm{d}\Omega} (\theta ,0 {}^{\circ },E_{\mathrm{in}})}.
\end{eqnarray*}

The asymmetry, another observable quantity proportional to the DOP of the incident $\gamma$ rays, is defined as


(6) \begin{eqnarray*}
&& A(\theta ,E_{\mathrm{in}})\\
&&=\frac{ {\frac {\mathrm{d}\sigma }{\mathrm{d}\Omega } (\theta ,90 {}^{\circ },E_{\mathrm{in}})} - {\frac{ \mathrm{d}\sigma } {\mathrm{d}\Omega }} (\theta ,0 {}^{\circ },E_{\mathrm{in}})}
{{\frac{\mathrm{d}\sigma } {\mathrm{d}\Omega }(\theta ,90 {}^{\circ },E_{\mathrm{in}})} + {\frac{\mathrm{d}\sigma } {\mathrm{d}\Omega } (\theta ,0 {}^{\circ },E_{\mathrm{in}})}},
\end{eqnarray*}

and can also be derived from the PVR.

The theoretical model establishes an effective energy threshold for this method at $E_\mathrm{in} < 4 \,\mathrm{MeV}$. This limit arises because both the measurable scattering asymmetry and the optimal detector angle $\theta _{\mathrm{max}}$—which are essential for precise quantification—decrease with increasing $\gamma$-ray energy, as described by the Compton cross section (see Section S3.2) [9,35,36].

To obtain the theoretically expected asymmetry $A_{\mathrm{th}}$, a Monte Carlo simulation was performed using GEANT4 [48], incorporating realistic beam parameters together with inputs such as the target and detector dimensions and positions (see Sections S2.2 and 3.2). In the polarization experiment, the differential cross section is represented by the azimuthal distribution of detected counts, which is fitted using


(7) \begin{eqnarray*}
N(\phi )=P_{0}-P_{1}\cos ^2\frac{\pi (\phi -P_{2})}{180},
\end{eqnarray*}

where ${P_0}$ and $P_1$ are used to calculate the experimental asymmetry $A_{\mathrm{exp}}$ and its uncertainties, and $P_2$ is the phase shift (in degrees) associated with the AOP. Finally, the DOP of the incident $\gamma$ rays is obtained as [23]


(8) \begin{eqnarray*}
\mathrm{DOP} = \frac{A_{\mathrm{exp}}}{A_{\mathrm{th}}}=\frac{P_{1}}{(2P_{0}-P_{1})A_{\mathrm{th}}}.
\end{eqnarray*}

A precisely controlled three-hole collimator (with diameters of $1 \,\mathrm{mm}$, $2 \,\mathrm{mm}$ and $3 \,\mathrm{mm}$) was used to scan the transverse two-dimensional (2D) distribution of polarized $\gamma$ rays; only the 1-mm-diameter aperture was employed in this experiment. At each scanning position, the collimated $\gamma$ rays irradiated a cylindrical Ta target of 99.9% purity with a diameter of $6 \,\mathrm{mm}$ and a thickness of $10 \,\mathrm{mm}$. The secondary photons scattered from the Ta target were then measured using an array of eight $\mathrm{LaBr}_{3}\mathrm{(Ce)}$ detectors, arranged azimuthally symmetrically around the Ta target at a radial distance of $19.8 \,\mathrm{cm}$. The array geometry is shown in Fig. 1a.

Before measuring the azimuthal distributions, the collimator positions were scanned to determine the center point of the $\gamma$-ray beam spot. Combined with the $\gamma$-ray spot data obtained from the $\gamma$-ray imaging system, 49 scanning points were arranged symmetrically along four radial directions separated by $45 {}^{\circ }$, as illustrated in Fig. 3 below. At every scanning point, the flux and energy of the incident polarized $\gamma$ rays were measured using a BGO detector placed in the beam dump (see Section S4).

**Figure 2.**
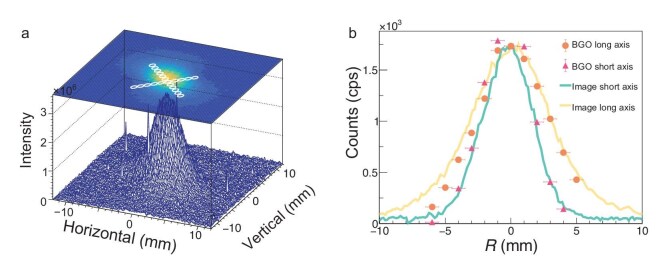
(a) Measured spatial distributions of scattered polarized $\gamma$ rays obtained using the $\gamma$-ray imaging system and collimator scanning. The three-dimensional spatial distribution is reconstructed from the $\gamma$-ray imaging data and projected onto the top view, where the white circles indicate the positions from collimator scanning. (b) Comparison of the scattered polarized $\gamma$-ray intensities along the long and short axes obtained from the $\gamma$-ray imaging system and collimator scanning. The image data are scaled and smoothed using the ROOT kernel algorithm (k5a, repeated twice). The scale factor is defined as the ratio of the maximum value of the image data to the BGO-measured value at the centre (R0). The error bars of the BGO data, which include statistical uncertainties and a 2% unfolding-algorithm uncertainty, are almost invisible.

**Figure 3.**
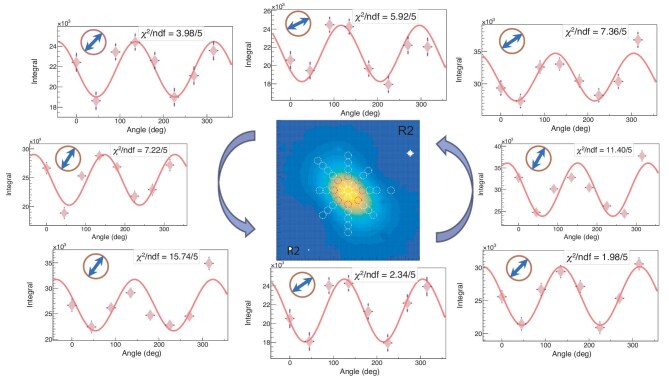
The profile of the $\gamma$ rays without the collimator is visualized at the center, where all scanning points are indicated by white circles, and the points corresponding to the surrounding azimuthal distributions are highlighted by red circles. The azimuthal distribution of the secondary photons scattered from the Ta target is shown in the corresponding direction when the $1 \,\mathrm{mm}$ collimator is aligned at a position $2 \,\mathrm{mm}$ away from the center of the $\gamma$-ray beam (R2). The diamond symbols represent experimental data obtained with the $\mathrm{LaBr}_{3}\mathrm{(Ce)}$ detectors, the solid line shows the fitting result, and the fitting parameter $\chi ^2$ and the AOP are indicated by the blue double-headed arrow.

### Intensity azimuthal distribution

Figure 2a shows the measured spatial distributions of the incident $\gamma$ rays, acquired using the $\gamma$-ray image system. This system relies on scintillator fluorescence captured by a CCD camera, offering direct 2D $\gamma$-ray imaging capability that is essential for spatially polarization analysis. In contrast, previous studies [22,24–26] have employed pixelated detectors, such as MiniPIX and CdTe image sensors, which provide only beam-profile information. Although the optical response of scintillators and system scattering effects can complicate absolute intensity calibration [49–51], the imaging system delivers indispensable spatial information for beam alignment and AOP mapping.

For accurate intensity measurements, we complemented the $\gamma$-ray imaging system with a BGO detector, which provides a well-understood and calibrated response to $\gamma$ rays, enabling direct and reliable quantification of the intensity—a capability that has been extensively validated in prior experiments [49–51]. This hybrid approach ensures comprehensive spatial mapping and quantification, advancing beyond methods that rely solely on pixelated detectors. The comparative analysis in Fig. 2b quantifies this difference along the beam-spot axes. To our knowledge, this represents the first systematic measurement of linearly polarized $\gamma$-ray intensity distributions using scanned BGO detection in combination with a $\gamma$-ray imaging system.

These results clearly show that the intensity distribution of the $\gamma$ rays is not continuously rotationally symmetric but is instead ‘pinched’ along the AOP of the laser beam. This behaviour is consistent with prior investigations at HI$\gamma$S [23] and NewSUBARU [22] under $180 {}^{\circ }$ backward ICS, as well as at UVSOR-III under $90 {}^{\circ }$ slant ICS [24].

### AOP distribution

A typical secondary scattering energy spectrum measured with a $\mathrm{LaBr}_{3}\mathrm{(Ce)}$ detector is shown in Fig. 1b. Fine geometric corrections based on Monte Carlo simulations were applied to each $\mathrm{LaBr}_{3}\mathrm{(Ce)}$ detector at every measurement point. The uncertainties arise from fluctuations in the intrinsic efficiency, statistical errors and uncertainties associated with the fine geometry-correction errors (see Section S5.2).

Figure 3 shows representative azimuthal distributions measured using the 1-mm-diameter collimator positioned precisely $2 \,\mathrm{mm}$ away from the incident $\gamma$-ray beam center along four radial directions (R2). The red curves represent fits using Equation (7). The extracted AOP values consistently align with the short axis of the beam spot at $45 {}^{\circ }$.

**Figure 4.**
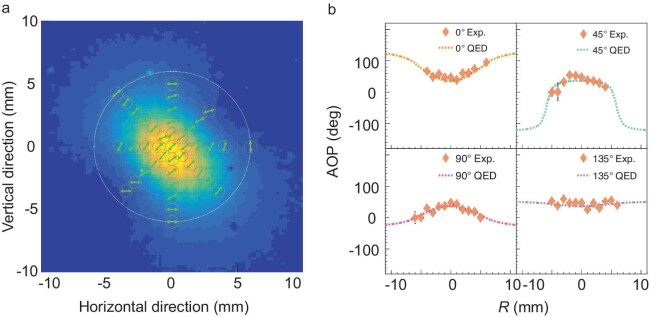
(a) AOP distribution, indicated by the green double-headed arrows, where the white dotted circle represents $1/\gamma _{L}$. (b) Comparison between the experimental results and calculations based on QED [52].

Further analysis of the intensities of secondary scattered photons across all scanning points yields the extracted AOP distribution shown in Fig. 4a. The measurement positions (small circular markers) correspond to collimator displacements relative to the $\gamma$-ray beam centre. At the beam center (the axis of the incident polarized $\gamma$ rays), the AOP strictly follows the short-axis direction at $45 {}^{\circ }$, in agreement with previous HI$\gamma$S results [23]. Within the proximal beam region ($\le$$2 \,\mathrm{mm}$ from the axis), the AOP remains uniformly distributed near $45 {}^{\circ }$. However, in lower-intensity peripheral regions, the AOP vectors exhibit tangential alignment relative to concentric circles.

Figure 4b compares the azimuthal distributions of the incident $\gamma$-ray AOP measured along different scanning directions with calculations based on the Stokes parameters (see Section S3.1). The measurements show good agreement with the theoretical predictions. Notably, under relativistic conditions, the scattered photons exhibit a distributed AOP within the $1/\gamma _{L}$ angular range, rather than maintaining a fixed polarization angle. This result demonstrates that experiments using linearly polarized $\gamma$ rays from laser Compton sources require careful selection of the polarization beam-spot size to ensure measurement fidelity.

### Asymmetry and DOP distribution

**Figure 5.**
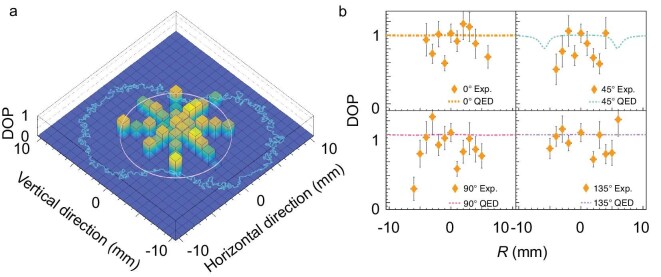
(a) DOP distribution, where the blue peanut-shaped contour indicates the $\gamma$-ray spot and the pink circle represents $1/\gamma _{L}$. (b) Comparison between the experimental results and calculations based on QED [52].

The average DOP of the $\gamma$-ray beam within the 1-mm-diameter collimator was obtained at all scanning points using Equation (8) and is shown in Fig. 5a. The uncertainty in the DOP arises mainly from statistical errors (less than 5.6% for the R0 data), efficiency-correction errors (less than 3.2%) and geometry-correction errors (less than 0.3%; see Section S5.2).

Near the beam center, the measured DOP is close to 1.0 ($1.03\pm 0.13$ at R0) whereas significant fluctuations are observed in the peripheral regions. Figure 5b compares experimental and theoretical DOP values along four scanning directions, showing good agreement.

Near-complete polarization transfer from the laser photons to the $\gamma$ rays is observed at the beam center for an incident angle of $45 {}^{\circ }$. This result demonstrates that $45 {}^{\circ }$ slant scattering provides an alternative pathway to generating high-DOP $\gamma$ rays beyond the traditional $180 {}^{\circ }$ backward ICS.

Chi [20] extended polarization-property calculations from $180 {}^{\circ }$ backward ICS to arbitrary slant geometries using a dipole-radiation model, predicting geometry-independent polarization transfer while noting degraded DOP and bandwidth with increasing collection angle. Similarly, QED-based simulations reported in [19] confirm this conclusion. Our measured peripheral DOP exhibits analogous spatial variations. Moreover, in previous work, under the assumption that the incident laser was 100% polarized, the DOP of $\gamma$ rays scattered at an angle of $1/\gamma _{L}$ was predicted to be close to 100%. However, under practical detection conditions, which involve averaging the polarization of photons over a solid angle, we found that the DOP decreases. Starting from the central point of the entire $\gamma$-ray spot, the AOP line can be extended and intersects the circle corresponding to the $1/\gamma _{L}$ scattering angle at two points. Near these two intersection points, the AOP of the photons exhibits rapid variations, analogous to the distribution of electric field lines around a point charge. Consequently, although the polarization degree of each individual photon is theoretically 1 according to polarization-transfer arguments, the average DOP within a 1 mm collimation aperture near these two regions is expected to decrease, as reflected by the two valleys in the QED-calculated curve shown in the upper-right panel of Fig. 5b. Unfortunately, the current experimental uncertainties prevent definitive quantitative validation of the theoretical DOP distributions in the peripheral regions, thereby limiting a complete characterization of polarization-transfer efficiency in slant scattering geometries. In high-energy ICS sources, the contribution of peripheral $\gamma$ rays becomes non-negligible owing to collimator limitations and could introduce systematic errors in polarization-sensitive measurements. This highlights the need for higher-precision polarization measurements in slant ICS experiments.

## CONCLUSION

Addressing a critical knowledge gap in the polarization properties of photons scattered from relativistic electrons, we have conducted the first systematic measurement of the spatial polarization distribution of $\gamma$ rays generated via $45 {}^{\circ }$ slant inverse Compton scattering between linearly polarized $0.117 \,\mathrm{eV}$ ($\lambda$ = $10.64 \,\mathrm{{\mu }m}$) $\mathrm{CO}_{2}$ laser photons and $3.5 \,\mathrm{GeV}$ relativistic electrons. Leveraging the fundamental principle that linearly polarized $\gamma$ rays induce asymmetric angular distributions in secondary scattered photons, we systematically measured intensity profiles, the AOP, asymmetry parameters and the DOP. The resulting linearly polarized $\gamma$-ray beam exhibits an asymmetric intensity profile, consistent with $180 {}^{\circ }$ backward ICS observations at HI$\gamma$S [23] and NewSUBARU [22], with the beam spot ‘pinched’ along the laser AOP direction. At the beam center, the AOP strictly aligns with the short-axis direction at $45 {}^{\circ }$ (consistent with HI$\gamma$S observations [23]), and the DOP measures almost 1.0. Near the beam axis, the AOP remains uniformly distributed around $45 {}^{\circ }$, whereas the peripheral regions exhibit tangential alignment relative to concentric circles and complex DOP variations.

These results demonstrate equivalent polarization-transfer efficiency near the beam axis for $45 {}^{\circ }$ slant ICS and traditional $180 {}^{\circ }$ backward ICS, thereby establishing slant scattering as a viable alternative for generating high-DOP $\gamma$ rays and significantly expanding the parameter space for polarized $\gamma$-ray sources. The uncertainties in the present experiment limit precise quantification of the DOP in the peripheral regions, precluding a full characterization of polarization-transfer efficiency in slant geometries. Future investigations are planned at SLEGS to achieve enhanced measurements of the polarization distribution.

Furthermore, this work lays the foundation for a distinct and complementary approach to beam modulation. By employing a precision-controlled collimator for spatial filtering, key beam properties—including energy, monochromaticity, intensity and polarization—can be selectively tuned in a simple, cost-effective and geometry-independent manner, without modulating the electron parameters, laser parameters or interaction geometry. This ‘post-generation’ spatial-filtering method could significantly simplify the design and operation of future ICS sources for applications requiring tailored beam profiles.

## Supplementary Material

nwag073_Supplemental_File
