# Supplementary material for: First systematic experimental 2D mapping of linearly polarized γ-ray polarimetric distributions in relativistic Compton scattering
Source: Natl Sci Rev. 2026 Feb 9;13(7):nwag073. doi: 10.1093/nsr/nwag073 (PMC13102005; doi:10.1093/nsr/nwag073)
Supplement: nwag073_Supplemental_File [file nwag073_supplemental_file.pdf]

Supplementary Materials of the manuscript
First systematic 2D polarimetric mapping of linear polarized γ-ray spatial distribution in relativistic Compton scattering

Kaijie Chen,[1,2] Xiangfei Wang,[3,4] Hanghua Xu,[2,4,*] Gongtao Fan,[2,3,4,†] Zhenwei Wang,[3,4] Zirui Hao,[2] Longxiang Liu,[2] Yue Zhang,[2] Sheng Jin,[3,4] Zhicai Li,[2] Pu Jiao,[2] Qiankun Sun,[3,4] Mengdie Zhou,[2] Yulong Shen,[2] Mengke Xu,[3,4] Chang Yang,[2] Jiawen Ding,[3,4] Hongwei Wang,[2,3,4] Wenqing Shen,[2,4] and Yugang Ma[5,1,6,‡]

[1]*ShanghaiTech University, Shanghai 201210, China*

[2]*Shanghai Advanced Research Institute, Chinese Academy of Sciences, Shanghai 201210, China*

[3]*Shanghai Institute of Applied Physics, Chinese Academy of Sciences, Shanghai 201800, China*

[4]*University of Chinese Academy of Sciences, Beijing 100049, China*

[5]*Institute of Modern Physics, Fudan University, Shanghai 200433, China*

[6]*School of Physics, East China Normal University, Shanghai 200062, China*

(Dated: January 17, 2026)

In this supplementary materials, we provide detailed information on the following:

- beamline Shanghai Laser Electron Gamma Source (SLEGS) 1: especially the determination of the DOP of the incident laser;
- experimental setup 2;
- the theoretical calculation for slant Inverse Compton Scattering (ICS) process and Monte Carlo simulation for second Compton scattering 3;
- measurement procedures 4;
- data processes and summary 5: especially error propagation and evaluation.

Notably, the definitions and explanations of symbols in some formulas are consistent with the main document. This file serves solely as additional content to the main document and is not independent and integrated.

1. SLEGS AT SSRF

SLEGS, one of the beamline at Shanghai Synchrotron Radiation Facility (SSRF), delivers quasi-monoenergetic γ-rays with tunable energy [1, 2], high intensity [3, 4], and high polarization [5, 6]. Its core components include a laser system, interaction chamber [7], collimation system [8, 9], and detectors [2, 10], etc [11]. Below is a concise introduction of the laser system relevant to this work. Further details can be found in the references.

1.1. Laser system

The simplified diagram of the laser optical transport system is shown in Fig.1 (a) of the main document, where M_{1-4} are plane mirrors for modulating laser propagation direction and f_1 is a convex lens for focusing laser photons [7]. An optical path for laser's DOP measurements is built. The incident laser go through a Thin-Film Polarizer (TFP), and then enter a power meter. The TFP can split a laser beam into two parts with orthogonal

* xuhh@sari.ac.cn
† fangt@sari.ac.cn
‡ mayugang@fudan.edu.cn

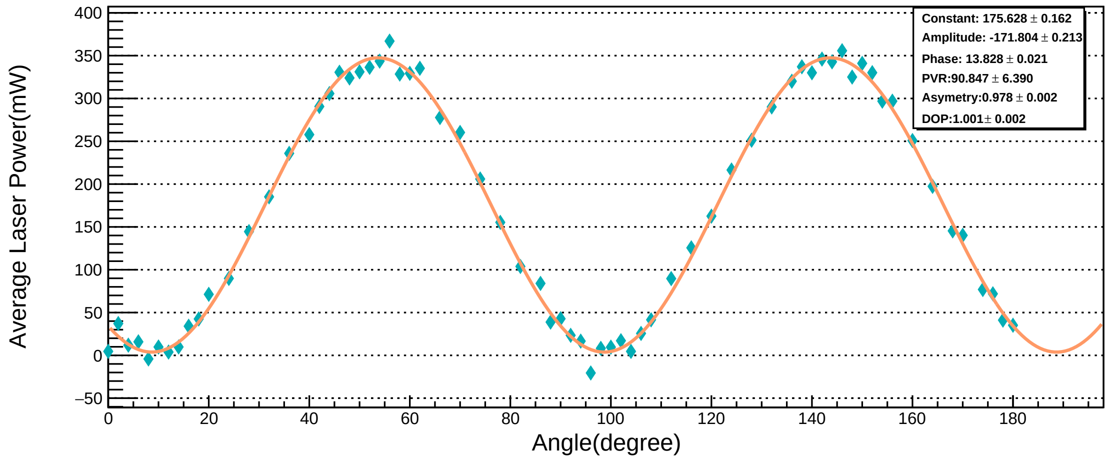

FIG. 1. The average power was measured as a function of different AOPs. The blue diamond markers represent the experimental data subtracted the background and the red solid lines represent the fitting results.

polarizations, specifically, s-polarized reflected and p-polarized transmitted components. A $\lambda/2$ waveplate is set upstream of TFP to change the angle of polarization (AOP) of linear polarized laser. Ideally, when the laser is incident at a Brewster angle relative to the TFP, the maximum intensity I_{max} is 98.6%, while the minimum intensity I_{min} is 1.14%. In other words, when a 100% linearly polarized laser is measured using the TFP under ideal geometry, the optimal asymmetry $A = \frac{I_{\text{max}}-I_{\text{min}}}{I_{\text{max}}+I_{\text{min}}}$ is only 97.71%. For each 4° step rotation of the $\lambda/2$ waveplate, the average power was measured over a 90 s interval. Near the signal maxima and minima, the step size was reduced to 2° to achieve finer scanning resolution. The background was also measured, when laser was powered off. The results subtracted background are shown in Fig. 1 and are fitted with the formula $A + B\sin[4(\phi + \phi_0)]$. The experimental asymmetry is 0.978±0.002. The ratio of 0.978 to 0.9771 is 1.001±0.002, which implies that the DOP of the incident laser is almost 100%.

2. EXPERIMENT SETUP

2.1. Alignment

In this experiment, we used two collimators [8, 9] manipulated by the five-dimensional motion stages offering three translational degrees of freedom (X/Y/Z linear positioning)

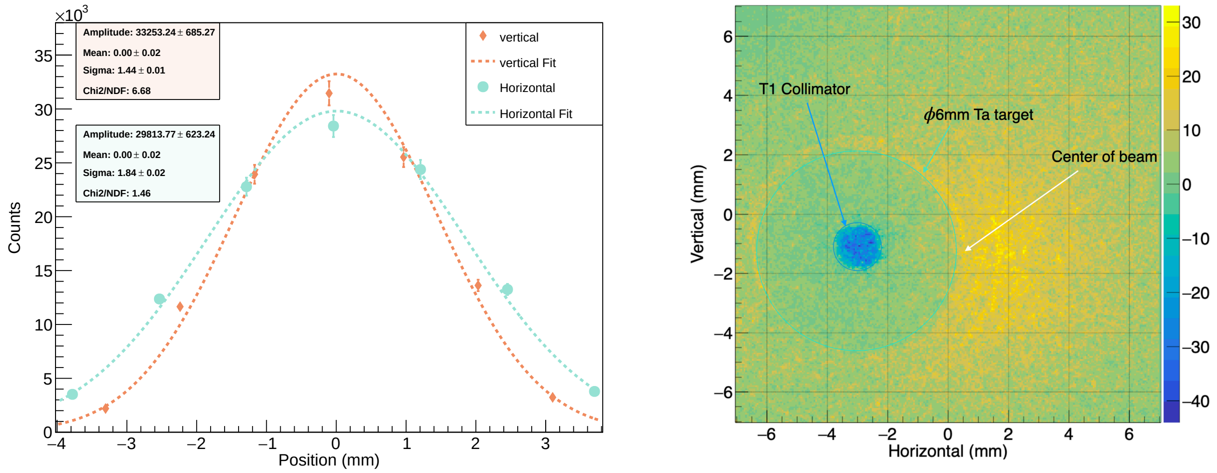

FIG. 2. The fluxes as a function of the positions of the three-hole collimator along horizontal and vertical direction respectively (in the left panel). The image (in the right panel) measured by a MiniPIX detector when the γ-rays go through the ϕ20 mm coarse collimator and the target is subtracted the image measured when the γ-rays go through the ϕ1 mm three-hole collimator at R3D180° point.

and two rotational axes (θ and ϕ). One is the coarse collimator with a diameter of 20 mm, approximately 18 m away from the interaction point, and the other is the three-hole collimator, approximately 36 m away from the interaction point. When the three-hole collimator is removed, we first adjust the position of the coarse collimator to ensure that the γ-rays beam spot within a half dispersion angle of $2/\gamma$ is completely exposed, which is estimated by the Gamma Spot Monitor (GSM) [11], and the MiniPIX detector. Then the three-hole collimator is set into the optical path. The flux of γ-rays after two-stage collimation is measured by the BGO detector [2], in diameter of 76 mm and in length of 200 mm. The flux is a function of the position of the three-hole collimator [1], like a parabola as shown in the left panel of Fig. 2. When the flux reaches the maximum value, the corresponding position of the three-hole collimator is the initial scan point called the R0 point. All scanning points are based on R0 as the reference point. For instance, Rx (x is an Arabic number) denotes a scan point located x millimeters away from R0.

The position of the 6 mm diameter Ta target, which is mounted on a 3D printed frame, can also be adjusted by the five-dimensional motion stage. When γ-rays go through the ϕ 20 mm coarse collimator and the Ta target, their relative position can clearly be seen in

the image captured by the MiniPIX detector. Therefore, controlling motion stage and using image feedback ensures that the three-hole collimator moves to the correct position and that the γ-rays hit the center of the target with an accuracy of 0.3 mm.

2.2. The $LaBr_3$(Ce) detector array

TABLE I. The initial spatial arrangement and intrinsic efficiency of each $LaBr_3$(Ce) detector. The (r, θ, ϕ) is in laboratory coordinate system and θ is the angle between γ-rays beam propagation direction and the detector. The initial geometry deviations of each $LaBr_3$(Ce) detector are the third, fourth and fifth columns in the Cartesian coordinate system. The γ-rays is positive Z direction.

ID	(r, θ, ϕ)	ΔX	ΔY	ΔZ	N_{BG}	ε_1	σ_1	ε_2	σ_2
NO.1	19.8 cm, 40°, 0°	4.323 mm	−0.029 mm	0.532 mm	528 cps	38.9%	0.4%	35.7%	0.2%
NO.2	19.8 cm, 40°, 45°	7.698 mm	3.032 mm	0.080 mm	592 cps	38.9%	0.4%	35.2%	0.4%
NO.3	19.8 cm, 40°, 90°	4.981 mm	0.644 mm	0.508 mm	516 cps	38.9%	0.4%	35.7%	0.2%
NO.4	19.8 cm, 40°, 135°	4.317 mm	−2.083 mm	2.853 mm	509 cps	39.5%	0.4%	35.9%	0.3%
NO.5	19.8 cm, 40°, 180°	4.073 mm	−1.853 mm	1.219 mm	534 cps	39.6%	0.5%	35.6%	0.3%
NO.6	19.8 cm, 40°, 225°	5.725 mm	−0.784 mm	0.362 mm	523 cps	39.2%	0.1%	36.0%	0.3%
NO.7	19.8 cm, 40°, 270°	−14.409 mm	2.486 mm	1.838 mm	499 cps	39.4%	0.4%	35.6%	0.2%
NO.8	19.8 cm, 40°, 315°	0.194 mm	−3.298 mm	−0.528 mm	548 cps	39.2%	0.2%	36.1%	0.2%

The array, consisting of eight $LaBr_3$(Ce), measured azimuthal distribution of secondary scattering γ-rays from a Ta target. Each $LaBr_3$(Ce) detector was installed on an aluminum bracket and the azimuthal angle between adjacent two detectors is 45°, as shown in Fig. 4. The geometry of the array and performance are shown in Table I. (r, θ, ϕ) denotes the spatial position of the $LaBr_3$(Ce) detector. N_{BG} is the rate of background including natural radiation and intrinsic radiation from ^{138}La and ^{227}Ac decay chain of $LaBr_3$(Ce) crystal. ε_1 and ε_2 are the efficiencies of 1173 keV and 1332 keV, respectively. The efficiency calibration is performed with all $LaBr_3$(Ce) detectors simultaneously irradiated by a ^{60}Co radioactive source at a distance of 100 cm. The distance is chosen long enough to suppress the influence of geometric receiving angle. Each detector is shielded by a low-background lead brick to

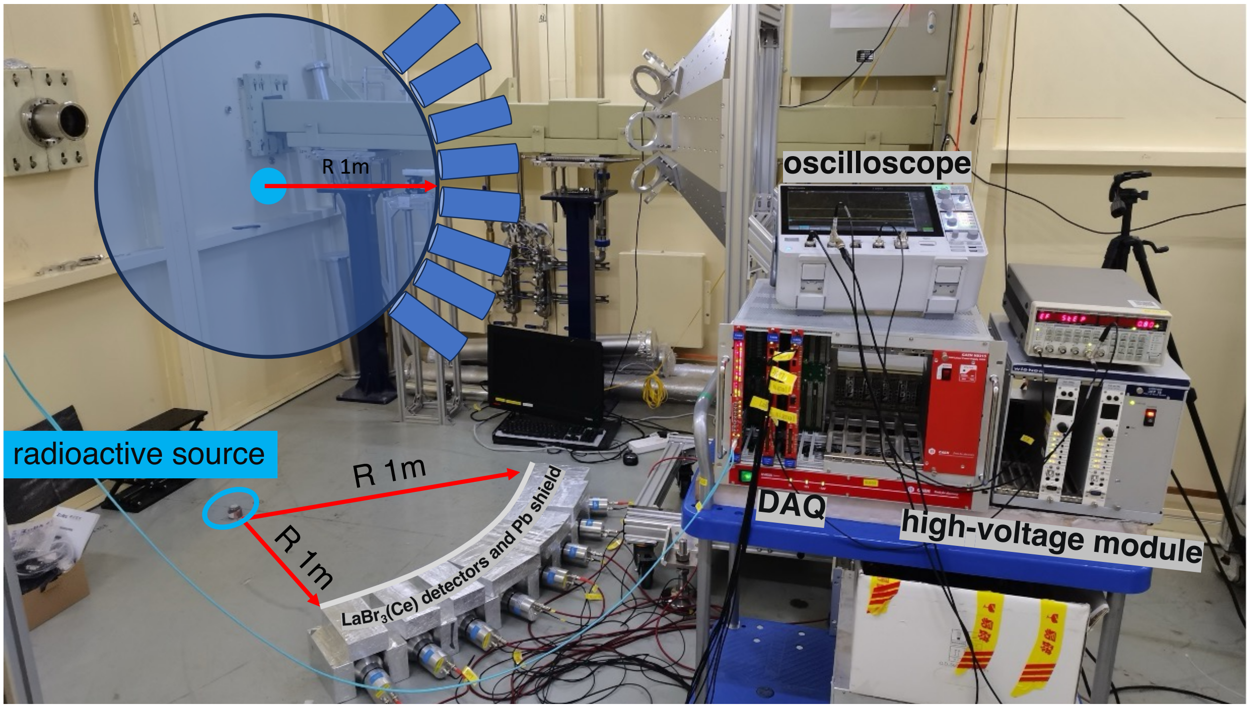

FIG. 3. The setup for calibration experiment. All detectors shielded with lead bricks are placed 1 meter away from the radioactive source.

suppress the ambient background radiation. The setup is shown in Fig. 3. The relative fluctuation of the efficiencies σ_i are evaluated over a time span of up to 16 h and give in columns 8 and 10 of Table I. From the results, it can be seen that the efficiency and corresponding fluctuations of each detector are very close, which is conducive to restoring the actual scattered photon angular distribution.

The asymmetry of Compton scattering of about 3 MeV γ-rays generated at the 45° slant Inverse Compton scattering (ICS) is less than 0.3 as shown in Fig. 7. Therefore, the geometric configuration of the detectors and target is a critical influencing factor. Throughout the experiment, the spatial positions of the detectors are fixed. The geometry of each detector was measured with a high accuracy of 28 µm using a portable measurement arms from the FARO Quantum X FaroArm Series portfolio, and was digitally modelled with the CAM2 software [12, 13]. The initial position deviations ΔX, ΔY and ΔZ for each $LaBr_3(Ce)$ detector are listed in columns 3, 4 and 5 of Table I

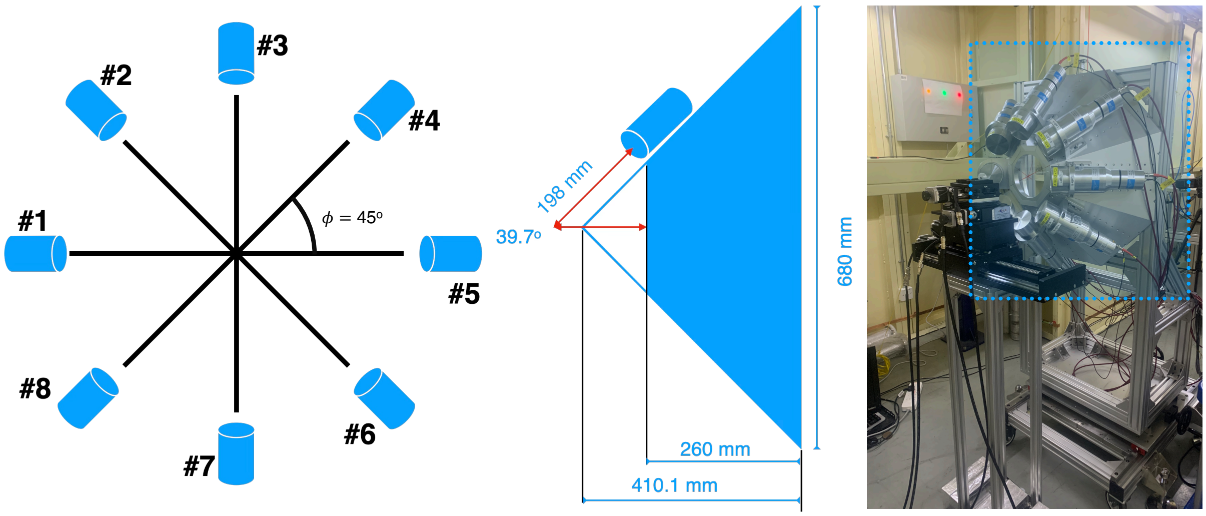

FIG. 4. The setup for this polarization experiment: beam propagation direction view (left panel); side view (middle panel) and 3D real photo (right panel).

3. THEORETICAL CALCULATION AND MONTE CARLO SIMULATIONS

This study involves two Compton scattering processes:

- 45° slant ICS between 0.117 eV laser photons and 3.5 GeV relativistic electrons, which is the process of generating SLEGS γ-rays beam;
- secondary scattering of SLEGS γ-rays with a target.

3.1. Introduction of Stokes parameters

Let's consider a generalized form of light propagating along the z direction within the framework of classical electrodynamics. The transverse components are given by

$$\begin{cases} E_x(z,t) = E_{0x}\cos(\tau + \delta_x) \\ E_y(z,t) = E_{0y}\cos(\tau + \delta_y) \end{cases} \tag{1}$$

where $\tau = \omega t - kz$, E_{x0} and E_{y0} are maximum amplitudes,and δ_x and δ_y are the phases. Expand the trigonometric functions, and hence

$$\begin{cases} \frac{E_x}{E_{0x}}\sin\delta_y - \frac{E_y}{E_{0y}}\sin\delta_x = \cos\tau\sin(\delta_y - \delta_x) \\ \frac{E_x}{E_{0x}}\cos\delta_y - \frac{E_y}{E_{0y}}\cos\delta_x = \sin\tau\sin(\delta_y - \delta_x) \end{cases} \tag{2}$$

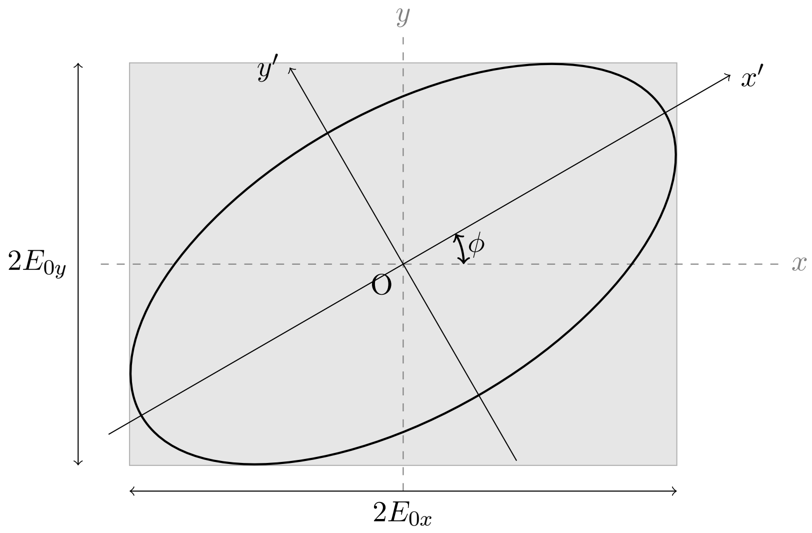

FIG. 5. An elliptically polarized wave

Square and add as follows

$$\frac{E_x^2}{E_{0x}^2} + \frac{E_y^2}{E_{0y}^2} - 2\frac{E_x E_y}{E_{0x}E_{0y}}\cos\delta = \sin^2\delta \tag{3}$$

where $\delta = \delta_y - \delta_x$. this equation 3 represents an ellipse and is shown as Fig. 5. The angle of rotation ϕ, representing the angle of the major axis of the ellipse relative to the laboratory coordinate system, relates the following equation

$$\tan 2\phi = \frac{2E_{ox}E_{0y}\cos\delta}{E_{0x}^2 - E_{0y}^2} \tag{4}$$

Considering the observables of the optical field from a experiment, we must take an average over the time of observation. The equation 3 is rewrite as

$$\frac{\langle E_x(t)^2\rangle}{E_{0x}^2} + \frac{\langle E_y(t)^2\rangle}{E_{0y}^2} - 2\frac{\langle E_x(t)\rangle\langle E_y(t)\rangle}{E_{0x}E_{0y}}\cos\delta = \sin^2\delta \tag{5}$$

where

$$\langle E_i(t)E_j(t)\rangle = \lim_{T\to\infty}\frac{1}{T}\int_0^T E_i(t)E_j(t)\,dt \quad i,j = x,y. \tag{6}$$

The Average values are

$$\langle E_x^2(t)\rangle = \frac{1}{2}E_{0x}^2 \tag{7}$$

$$\langle E_y^2(t)\rangle = \frac{1}{2}E_{0y}^2 \tag{8}$$

$$\langle E_x(t)E_y(t)\rangle = \frac{1}{2}E_{0x}E_{0y}\cos\delta \tag{9}$$

Substituting equation 7, 8 and 9 into 5, yields

$$4E_{0x}^2 E_{0y}^2 - (2E_{0x}E_{0y}\cos\delta)^2 = (2E_{0x}E_{0y}\sin\delta)^2 \tag{10}$$

and reorganizes as

$$(E_{0x}^2 + E_{0y}^2)^2 - (E_{0x}^2 - E_{0y}^2) - (2E_{0x}E_{0y}\cos\delta)^2 = (2E_{0x}E_{0y}\sin\delta)^2 \tag{11}$$

Introduce the Stokes parameter ξ_i as

$$\begin{cases} \xi_0 = E_{0x}^2 + E_{0y}^2 \\ \xi_1 = 2E_{0x}E_{0y}\cos\delta \\ \xi_2 = 2E_{0x}E_{0y}\sin\delta \\ \xi_3 = E_{0x}^2 - E_{0y}^2 \end{cases} \tag{12}$$

Therefore, equation 11 is rewrite as

$$\xi_0^2 = \xi_1^2 + \xi_2^2 + \xi_3^2. \tag{13}$$

For any state of polarized light, the Stokes parameters always satisfy the relation

$$\xi_0^2 \geq \xi_1^2 + \xi_2^2 + \xi_3^2. \tag{14}$$

The DOP for any state of polarization is defined by

$$\mathrm{DOP} = \frac{I_{\mathrm{pol}}}{I_{\mathrm{tot}}} = \frac{(\xi_1^2 + \xi_2^2 + \xi_3^2)^{1/2}}{\xi_0} \tag{15}$$

Similarly, equation 4 is rewrite as

$$\tan 2\phi = \frac{\xi_1}{\xi_3} \tag{16}$$

The equation 12 are the Stokes polarization parameters for a plane wave and were introduced into optics by George Gabriel Stokes in 1852 [14, 15]. The four Stokes polarization parameters ξ_i is the total intensity of light, the amount of linear +45° or -45° polarization, amount of right or left circular polarization and the amount of linear horizontal or vertical polarization, respectively. When the phase and amplitude satisfy special conditions, elliptical polarization evolve into a special polarization state as following:

- **Elliptical polarization:** Elliptically polarized light simultaneously possesses the ξ_1, ξ_2, and ξ_3 components.

- **Linear polarization:** When $\delta = 0$ or $\delta = \pi$, elliptical polarization degenerates into linear polarization. A fully linearly polarized photon possesses only the ξ_1 and ξ_3 components, with ξ_2 being zero. It dominates in our experimental configuration, particularly in the paraxial region.

- **Circular polarization:** When $\delta = \pm\frac{\pi}{2}$ and $E_{0x} = E_{0y}$, elliptical polarization degenerates into circular polarization. A fully circularly polarized photon possesses only the ξ_2 component, lacking ξ_1 and ξ_3.

The AOP, equivalently ϕ in Fig. 5 and equation 16, is a measurement quantity specific to linearly polarized gamma rays. For elliptically polarized light, since a linear polarization component still exists, the angular distribution of scattered light can still be detected.

Under the framework of quantum mechanics, the polarization state of a single photon can be described by a two-dimensional complex vector:

$$\boldsymbol{\Psi} = c_1\boldsymbol{\Psi_1} + c_2\boldsymbol{\Psi_2} \tag{17}$$

where $\boldsymbol{\Psi_1}$ and $\boldsymbol{\Psi_2}$ are orthogonal polarization states for a photon, and c_1^2 and c_2^2 yield the relative probabilities for a single photon in the states $\boldsymbol{\Psi_1}$ and $\boldsymbol{\Psi_2}$, respectively. The polarization vector $\boldsymbol{\Psi}$ acts as the wave function of the "spin part". The polarization of photon is determined by the complex amplitudes c_1 and c_2

$$\boldsymbol{\rho} = \begin{pmatrix} \rho_{11} & \rho_{12} \\ \rho_{21} & \rho_{22} \end{pmatrix} = \begin{pmatrix} c_1c_1^* & c_1c_2^* \\ c_2c_1^* & c_2c_2^* \end{pmatrix} \tag{18}$$

its components:

- ρ is a polarization density matrix, which is a 2×2 Hermitian matrix;

- The off-diagonal elements, complex numbers, represent the coherence or correlation between different polarization basis states.

  - Their real parts encode the information for ±45° linear polarization ξ_1;

  - Their imaginary parts encode the information for left/right circular polarization ξ_2.

- The trace of the matrix $\mathrm{Tr}(\boldsymbol{\rho}) = \rho_{11} + \rho_{22} = 1$, which represents the normalization of the total probability.

Introduce the Stokes parameters

$$\begin{cases} \xi_1 & = \rho_{12} + \rho_{21} \\ \xi_2 & = i(\rho_{12} - \rho_{21}) \\ \xi_3 & = \rho_{11} - \rho_{22} \end{cases} \tag{19}$$

and expand the density matrix as following

$$\boldsymbol{\rho} = \frac{1}{2} \begin{pmatrix} 1 + \xi_3 & \xi_1 - i\xi_2 \\ \xi_1 + i\xi_2 & 1 - \xi_3 \end{pmatrix} \tag{20}$$

Therefore, DOP and AOP are

$$\mathrm{DOP} = \frac{\sqrt{(\rho_{11} - \rho_{22})^2 + 4\rho_{12}\rho_{21}}}{\rho_{11} + \rho_{22}} \tag{21}$$

$$\mathrm{AOP} = \frac{1}{2} \arctan \frac{\xi_1}{\xi_3} = \frac{1}{2} \arctan \frac{\rho_{12} + \rho_{21}}{\rho_{11} - \rho_{22}} \tag{22}$$

The equation 20 serves as a bridge between the macroscopic measurement (Stokes parameters) and the microscopic quantum statistical description (density matrix). If we diagonalize the density matrix, its eigenvectors give the dominant "average" polarization direction in the beam, and its eigenvalues give the weight of the intensity in that direction.

For single photons, the degree of polarization is given by the above formula. However, for incoherent multiphotons, the formulas for calculating DOP and AOP differ slightly. Its formula is calculated as follows

$$\mathrm{DOP} = \sqrt{\langle \xi_1 \rangle^2 + \langle \xi_2 \rangle^2 + \langle \xi_3 \rangle^2} \tag{23}$$

$$\mathrm{AOP} = 1/2 \arctan(\langle \xi_1 \rangle / \langle \xi_3 \rangle) \tag{24}$$

Where $\langle \xi_i \rangle$ is the average Stokes parameters of all photons that is detected.

3.2. Process of slant ICS

According to the conservation of the 4-momentum, the 4-momenta before and after scattering are related by

$$p + k = p' + k', \tag{25}$$

where $p = (E_r/c, \vec{p})$ and $k = (E_p/c, \hbar\vec{k})$ are the 4-momenta of the electron and photon before scattering, respectively, and p' and k' are their 4-momenta after scattering. Using quantum electrodynamics (QED) theory [16, 17], the Compton scattering cross section in the Lorentz invariant form for unpolarized relativistic electrons scattering with polarized photons is given by

$$\begin{aligned}\frac{d\sigma}{dY d\phi_f} =& \frac{2r_e^2}{X^2}\left\{\left(\frac{1}{X}-\frac{1}{Y}\right)^2+\frac{1}{X}-\frac{1}{Y}+\frac{1}{4}\left(\frac{X}{Y}+\frac{Y}{X}\right)\right.\\&-(\xi_3+\xi_3')\left[\left(\frac{1}{X}-\frac{1}{Y}\right)^2+\frac{1}{X}-\frac{1}{Y}\right]\\&+\xi_1\xi_1'\left(\frac{1}{X}-\frac{1}{Y}+\frac{1}{2}\right)+\xi_2\xi_2'\frac{1}{4}\left(\frac{X}{Y}+\frac{Y}{X}\right)\\&\times\left(1+\frac{2}{X}-\frac{2}{Y}\right)+\xi_3\xi_3'\left[\left(\frac{1}{X}-\frac{1}{Y}\right)^2\right.\\&\left.\left.+\frac{1}{X}-\frac{1}{Y}+\frac{1}{2}\right]\right\},\end{aligned} \tag{26}$$

where r_e is the classical electron radius, ϕ_f is the azimuthal angle of the scattered photon, ξ_i and ξ_i' are Stokes parameters describing the incident and scattered photon polarizations in their local coordinate systems (x_e, y_e, z_e), respectively. In the fixed electron coordinate system, Stokes parameter of the incident photon are written as $\xi_1 = P_t \sin(2\tau - 2\phi_f), \xi_2 = P_c, \xi_3 = -P_t \cos(2\tau - 2\phi_f)$, P_t and P_c are the degree of linear and circular polarizations of incident photon; X and Y are the Lorentz invariant variables defined as follows:

$$X = \frac{s-(mc)^2}{(mc)^2}, \quad Y = \frac{(mc)^2-u}{(mc)^2} \tag{27}$$

where s and u are the Mandelstam variables given by

$$s = (p+k)^2, \quad u = (p-k')^2. \tag{28}$$

If we assume

$$
\begin{cases}
\Phi_0 = \left(\frac{1}{X}-\frac{1}{Y}\right)^2 + \frac{1}{X} - \frac{1}{Y} + \frac{1}{4}\left(\frac{X}{Y}+\frac{Y}{X}\right) + \left[\left(\frac{1}{X}-\frac{1}{Y}\right)^2 + \frac{1}{X} - \frac{1}{Y}\right] P_t \cos(2\tau - 2\phi_f) \\
\Phi_1 = \left[\left(\frac{1}{X}-\frac{1}{Y}\right)^2 + \frac{1}{X} - \frac{1}{Y} + \frac{1}{2}\right] P_t \cos(2\tau - 2\phi_f) \sin 2\phi_f \\
\qquad - \left(\frac{1}{X} - \frac{1}{Y} + \frac{1}{2}\right) P_t \sin(2\tau - 2\phi_f) \cos 2\phi_f + \left[\left(\frac{1}{X}-\frac{1}{Y}\right)^2 + \frac{1}{X} - \frac{1}{Y}\right] \sin 2\phi_f \\
\Phi_2 = \frac{1}{4}\left(\frac{X}{Y}+\frac{Y}{X}\right)\left(1 + \frac{2}{X} - \frac{2}{Y}\right) P_c \\
\Phi_3 = \left(\frac{1}{X} - \frac{1}{Y} + \frac{1}{2}\right) P_t \sin 2\phi_f \sin(2\tau - 2\phi_f) + \left[\left(\frac{1}{X}-\frac{1}{Y}\right)^2 + \frac{1}{X} - \frac{1}{Y}\right] \cos 2\phi_f \\
\qquad + \left[\left(\frac{1}{X}-\frac{1}{Y}\right)^2 + \frac{1}{X} - \frac{1}{Y} + \frac{1}{2}\right] P_t \cos 2\phi_f \cos(2\tau - 2\phi_f)
\end{cases}
\tag{29}
$$

the Eq. 26 can be written as

$$
\frac{\mathrm{d}\sigma}{\mathrm{dYd}\phi_\mathrm{f}} = \frac{2r_e^2}{X^2}(\Phi_0 + \sum_{i=1}^{3} \Phi_i \xi_i^{det}) \tag{30}
$$

Where ξ_i^{det} is the polarization of the scattered photon selected by a detector. The Stokes parameters of scattered photon itself ξ_i' are given by

$$
\xi_i' = \frac{\Phi_i}{\Phi_0}, \quad i = 1, 2, 3 \tag{31}
$$

The final averaged Stokes parameters $\langle \xi_i' \rangle$ of the scattered photons can be obtained by integrating within the collimating aperture.

3.3. Secondary Scattering γ-rays from target

Using the Klein-Nishina formula, we compute the distribution of secondary scattered γ-rays from the target for arbitrary geometric configurations and incident γ-ray energies. Figure 6 and 7 present the calculated intensity, Peak-to-Valley Ratio (PVR) and asymmetry distribution.

Given the actual detector dimensions, geometric positions, energy distribution of the SLEGS generated γ-rays beam, and other interaction effects, Monte Carlo simulation provides a more direct approach than theoretical formulae. As a standard Monte Carlo

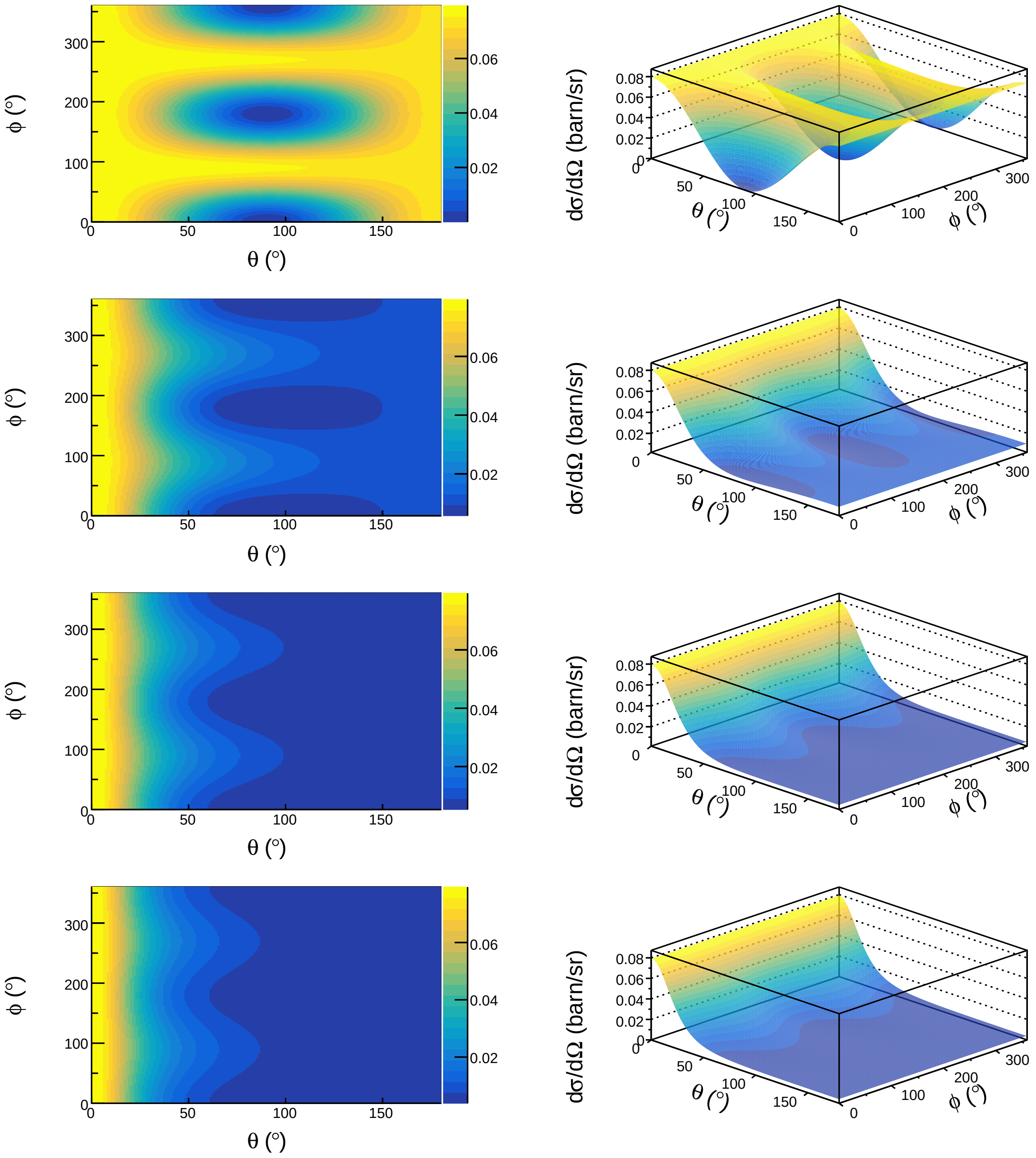

FIG. 6. The calculated intensity distribution of secondary Compton scattering γ-rays from Ta target based on the Klein–Nishina formula corresponding to 0.8 MeV, 1.6 MeV, 2.4 MeV and 3.2 MeV incident linear polarized γ-rays.

framework, the Geant4 toolkit [18] is used. It has been extended beyond the standard ElectroMagnetic (EM) physics processes to include the polarization-dependent processes. The extension implements a Stokes vector formalism for each particle, tracking the mean polarization state evolution across interactions by applying the matrix transformations

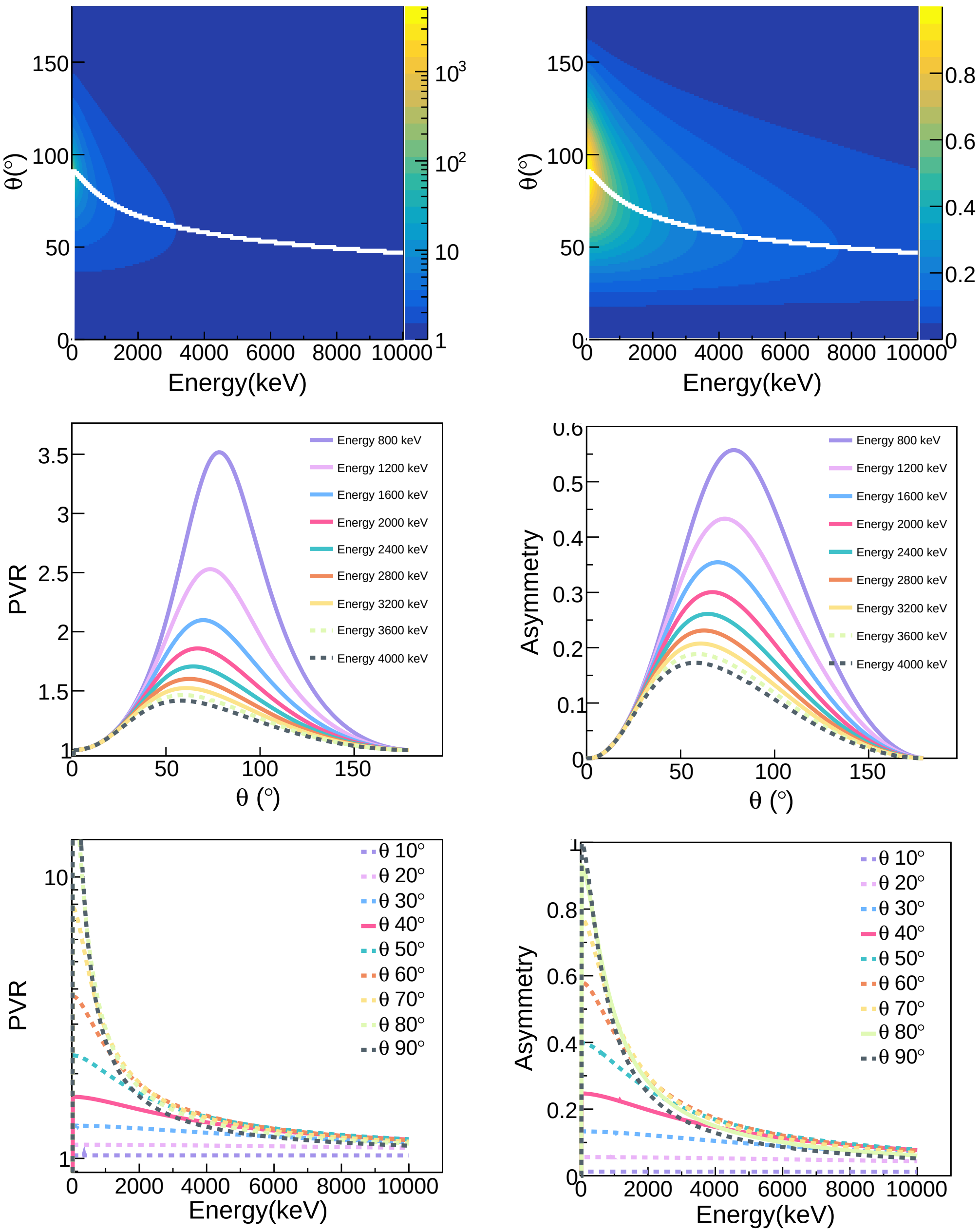

FIG. 7. The calculated PVR and asymmetry distribution of secondary Comtpon scattering γ-rays from Ta target based on the Klein–Nishina formula from 10 keV to 10 MeV incident linear polarized γ-rays. The white line represents the optimal scattering angle.

that incorporate cross-section, polarization transfer, and depolarization effects. Our simulation geometry replicates the experimental setup. We implemented a custom SLEGS-ParticleGun class, inheriting from `G4ParticleGun`, to generates the γ-rays with SLEGS-like energy spectra and specified polarization states. Crucially, to avoid discrepancies be-

tween theory and experiments for slant ICS, SLEGS-ParticleGun directly samples input experimental energy spectra rather than simulating slant ICS process itself. The classes `G4PolarizedPhotoElectric`, `G4PolarizedCompton`, and `G4PolarizedGammaConversion` for γ-rays interaction, `G4eMultipleScattering`, `G4PolarizedIonisation`, `G4PolarizedBremsstrahlung` and `G4PolarizedAnnihilation` for positron-electron interaction are invoked. These modules enable calculation of theoretical asymmetries.

3.4. Threshold of the detector

The impact of varying energy thresholds on azimuthal distributions and asymmetry parameters was evaluated via Monte Carlo simulations. As an example, the azimuthal distributions with different thresholds at R0 under ideal geometric conditions are presented in the left panel of Fig. 8, where the error bar only considers statistical error and the dotted lines are the fitting results. The threshold-dependent asymmetries are presented in the right panel of Fig. 8, with uncertainties calculated via error propagation from fitting parameters according to established methods [19]. After comprehensive evaluation, the energy range of 550 keV to 1.4 MeV was identified as optimal for azimuthal distribution measurements and asymmetry analysis.

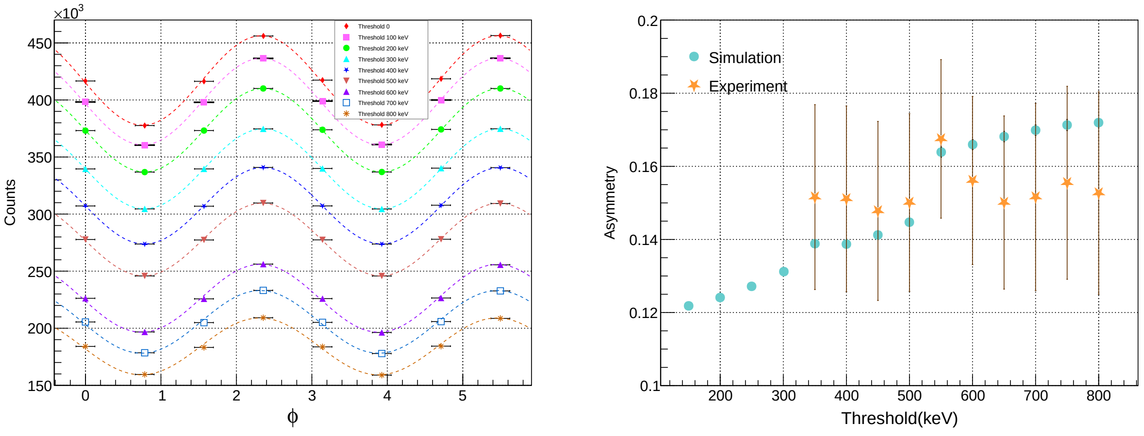

FIG. 8. The azimuthal distributions of the ideal geometry of the $LaBr_3(Ce)$ detectors array with different threshold at R0 in the left panel and the asymmetries as a function of threshold at R0 in the right panel.

3.5. Simulated PVR and Asymmetry

All simulated PVR and asymmetry $A_{\rm th}$ obtained using Geant4 toolkit are summarized in Table II.

TABLE II: Simulated PVR and Asymmetry

Direction	R	Monte Carlo simulations		Direction	R	Monte Carlo simulations	
		PVR	$A_{\rm th}$			PVR	$A_{\rm th}$
-	0 mm	1.390 ± 0.008	0.163 ± 0.002				
0°	1 mm	1.399 ± 0.008	0.167 ± 0.002	180°	1 mm	1.416 ± 0.014	0.172 ± 0.004
	2 mm	1.426 ± 0.008	0.175 ± 0.002		2 mm	1.429 ± 0.011	0.177 ± 0.002
	3 mm	1.463 ± 0.011	0.188 ± 0.002		3 mm	1.497 ± 0.010	0.199 ± 0.002
	4 mm	1.535 ± 0.019	0.211 ± 0.003		4 mm	1.570 ± 0.012	0.222 ± 0.002
	5 mm	1.622 ± 0.020	0.237 ± 0.003		5 mm	1.644 ± 0.017	0.243 ± 0.003
	6 mm	1.698 ± 0.014	0.259 ± 0.002		6 mm	1.730 ± 0.018	0.267 ± 0.002
45°	1 mm	1.390 ± 0.008	0.163 ± 0.002	225°	1 mm	1.399 ± 0.012	0.162 ± 0.003
	2 mm	1.429 ± 0.009	0.177 ± 0.002		2 mm	1.435 ± 0.010	0.179 ± 0.002
	3 mm	1.489 ± 0.011	0.196 ± 0.003		3 mm	1.500 ± 0.018	0.200 ± 0.003
	4 mm	1.547 ± 0.021	0.215 ± 0.005		4 mm	1.574 ± 0.010	0.223 ± 0.002
	5 mm				5 mm	1.665 ± 0.012	0.250 ± 0.002
	6 mm	1.756 ± 0.020	0.274 ± 0.003		6 mm	1.784 ± 0.019	0.282 ± 0.002
90°	1 mm	1.422 ± 0.010	0.174 ± 0.002	270°	1 mm	1.399 ± 0.013	0.167 ± 0.003
	2 mm	1.480 ± 0.014	0.194 ± 0.003		2 mm	1.453 ± 0.015	0.185 ± 0.003
	3 mm	1.577 ± 0.010	0.224 ± 0.002		3 mm	1.538 ± 0.014	0.212 ± 0.003
	4 mm	1.692 ± 0.021	0.257 ± 0.003		4 mm	1.655 ± 0.011	0.247 ± 0.002
	5 mm	1.792 ± 0.015	0.284 ± 0.002		5 mm	1.774 ± 0.028	0.279 ± 0.003
	6 mm	1.764 ± 0.021	0.276 ± 0.004		6 mm	1.799 ± 0.032	0.285 ± 0.004
135°	1 mm	1.416 ± 0.014	0.172 ± 0.003	315°	1 mm	1.397 ± 0.008	0.166 ± 0.002
	2 mm	1.491 ± 0.016	0.197 ± 0.004		2 mm	1.4450 ± 0.0009	0.192 ± 0.002
	3 mm	1.568 ± 0.022	0.221 ± 0.004		3 mm	1.515 ± 0.020	0.205 ± 0.004

Continued on next page

TABLE II – continued from previous page

Direction	R	Monte Carlo simulations		Direction	R	Monte Carlo simulations	
		PVR	A_{th}			PVR	A_{th}
	4 mm	1.664 ± 0.022	0.249 ± 0.003		4 mm	1.623 ± 0.021	0.238 ± 0.004
	5 mm	1.766 ± 0.014	0.277 ± 0.002		5 mm	1.743 ± 0.022	0.271 ± 0.003
	6 mm	1.794 ± 0.018	0.284 ± 0.002		6 mm		

3.6. Correction of the geometry

During experiment, the target was dynamically adjusted track the 1mm diameter hole of the three-hole collimator. The synchronous target-beam motion ensures on-target beam incidence, preventing artifacts in angular distribution measurements. Since the detectors are fixed, the solid angle subtended by the detector acceptance surface changes as the target moves, thus requires fine correction, particularly for data far from the central region. Accordingly, we carried out two steps Geant4 Monte Carlo simulations per data point, in which all the conditions were kept identical except for the geometry.

1. Ideal geometry: Simulating the scattered photons deposited in the $LaBr_3$(Ce) detectors array located in the nominal position with different the target and beam positions.

2. Actual geometry: Incorporating the positional deviations specified in Columns 3-5 of Table I.

Under ideal geometry, the move of the target and beam relative to detector array distorts the azimuthal distribution. Representative detector count ratios illustrating this effect are shown in Fig. 9, with the errors calculated according to the law of propagation of uncertainties and statistical errors.

TABLE III. The correct factor of the actual geometry of the $LaBr_3$(Ce) detectors array when the three-hole collimator is adjusted the R0.

ID	correct factor	ID	correct factor	ID	correct factor	ID	correct factor
No.1	0.9389 ± 0.0023	No.2	0.9900 ± 0.0025	No.3	1.0533 ± 0.0025	No.4	1.1058 ± 0.0025
No.5	1.0656 ± 0.0025	No.6	1.0136 ± 0.0026	No.7	0.9686 ± 0.0024	No.8	0.9273 ± 0.0022

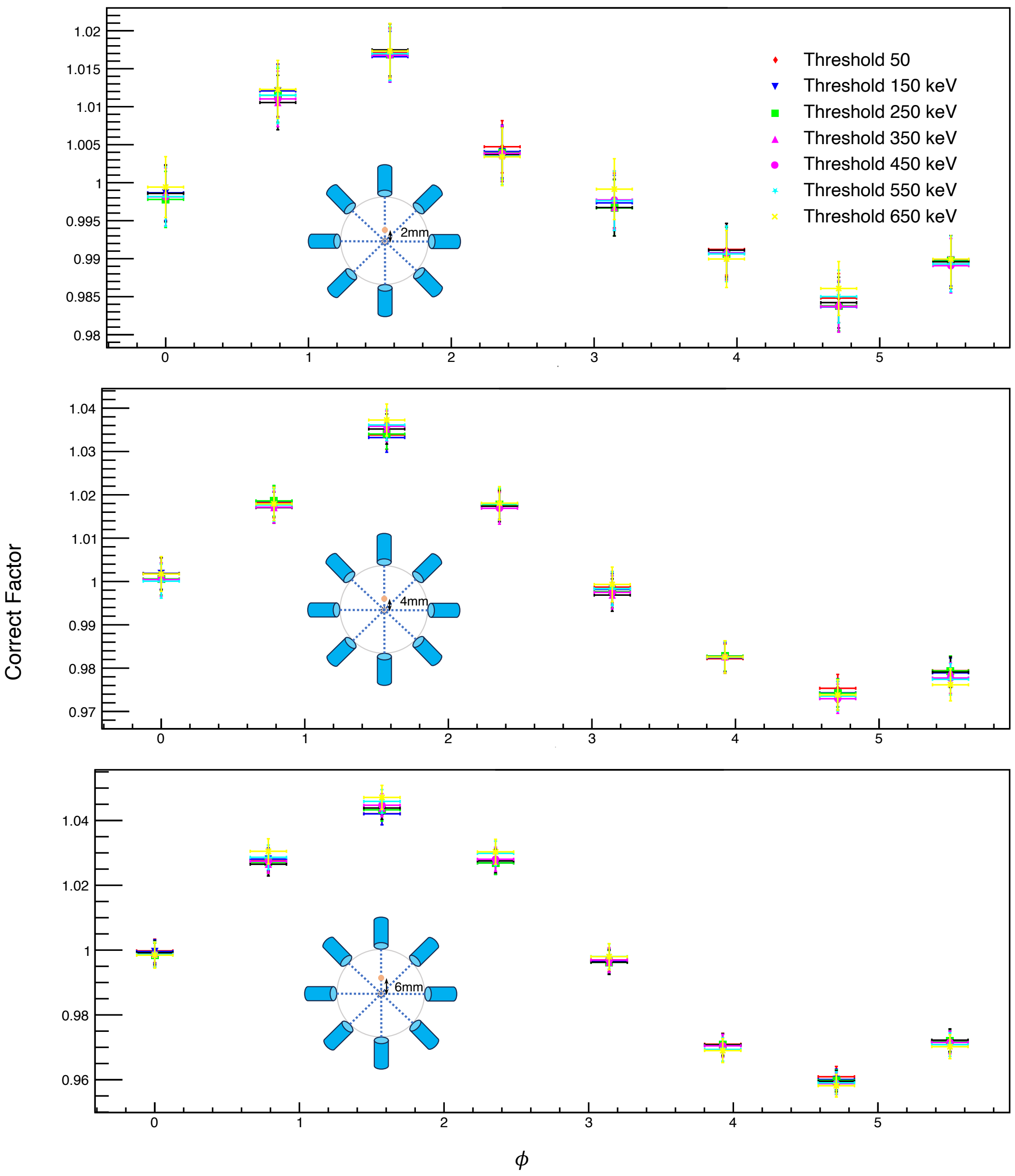

FIG. 9. The correct factor of the ideal geometry of the $LaBr_3$(Ce) detectors array with different threshold when the three-hole collimator is adjusted to R2 (in the top panel), to R4 (in the middle panel) and R6 (in the bottom panel) at 90° direction.

The correction factors of the actual geometry of the $LaBr_3$(Ce) detectors array at R0, are shown in Table III. The correction factor is defined as the ratio of counts per detector under actual geometry to those under ideal geometry at R0.

4. MEASUREMENT

4.1. Energy and Intensity of Incident Polarized γ-rays

The SSRF storage ring operates in top-up mode with current of 200 mA, while the laser utilizes a gated Continuous Wave (CW) mode triggered by a Radio Frequency (RF) enable signal with pulse repetition frequency of 1 kHz and pulse width of 50 µs. At every scanning point, the γ-rays were measured by a BGO detector placed in the beam dump. The bremsstrahlung and natural background of the spectra can be subtracted easily via the trigger signal. The time-normalized incident energy spectra obtained through direct unfolding [2] are shown in Fig. 10 for every scanning point.

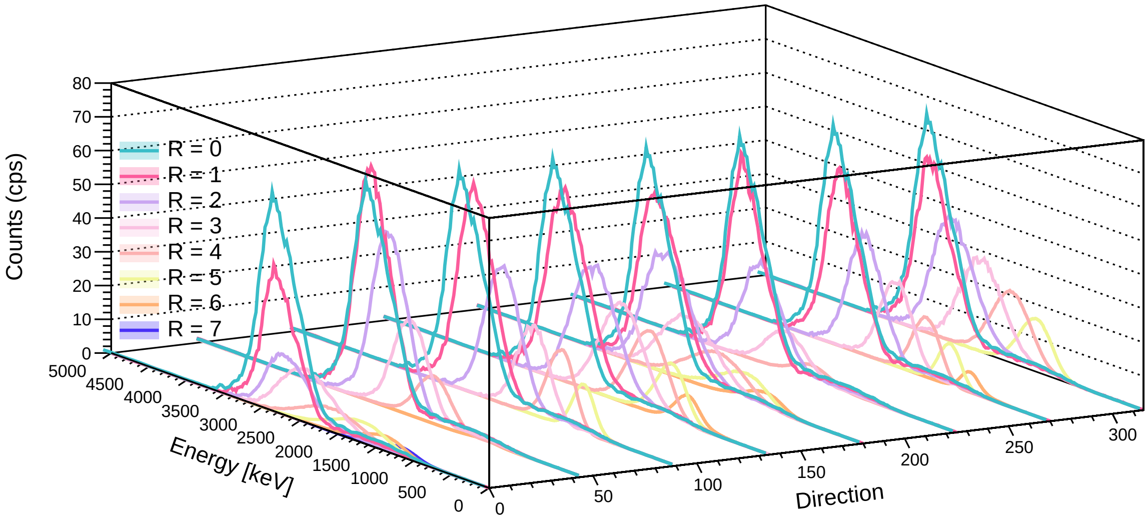

FIG. 10. The energy spectra of the incident γ-rays were measured by the BGO detector and unfolded by solving the BGO response matrix for each measurement point.

The intensity distribution is illustrated in Figure 2 in the main text. The detailed numbers are presented in Table IV.

TABLE IV: Incident γ-rays intensity measured by BGO.

Direction	R	Intensity	Direction	R	Intensity
-	0 mm	(1733.31 ± 18.72) cps			

Continued on next page

TABLE IV – continued from previous page

Direction	R	Intensity	Direction	R	Intensity
0°	1 mm	(1243.27 ± 13.45) cps	180°	1 mm	(1575.85 ± 17.00) cps
	2 mm	(753.13 ± 8.05) cps		2 mm	(1217.01 ± 13.15) cps
	3 mm	(799.94 ± 8.58) cps		3 mm	(750.01 ± 8.13) cps
	4 mm	(383.14 ± 4.11) cps		4 mm	(448.88 ± 4.88) cps
	5 mm	(279.66 ± 2.98) cps		5 mm	(282.14 ± 3.06) cps
	6 mm	(160.42 ± 1.71) cps		6 mm	(158.33 ± 1.73) cps
45°	1 mm	(1788.41 ± 19.25) cps	225°	1 mm	(1730.06 ± 18.69) cps
	2 mm	(1378.33 ± 14.74) cps		2 mm	(991.46 ± 10.66) cps
	3 mm	(737.34 ± 7.87) cps		3 mm	(406.88 ± 4.38) cps
	4 mm	(343.42 ± 3.60) cps		4 mm	(142.95 ± 1.53) cps
	5 mm			5 mm	
	6 mm	(13.87 ± 0.16) cps		6 mm	
90°	1 mm	(1628.75 ± 17.63) cps	270°	1 mm	(1551.07 ± 16.82) cps
	2 mm	(1070.90 ± 11.65) cps		2 mm	(1064.87 ± 11.59) cps
	3 mm	(605.77 ± 6.69) cps		3 mm	(703.36 ± 7.61) cps
	4 mm	(421.23 ± 4.59) cps		4 mm	(406.59 ± 4.43) cps
	5 mm	(224.72 ± 2.48) cps		5 mm	(233.29 ± 2.56) cps
	6 mm			6 mm	(104.52 ± 1.15) cps
135°	1 mm	(1690.18 ± 18.32) cps	315°	1 mm	(1608.07 ± 17.42) cps
	2 mm	(1220.60 ± 13.27) cps		2 mm	(1340.93 ± 14.44) cps
	3 mm	(885.22 ± 9.59) cps		3 mm	(1022.48 ± 10.95) cps
	4 mm	(623.05 ± 6.75) cps		4 mm	(693.11 ± 7.44) cps
	5 mm	(351.32 ± 3.86) cps		5 mm	(428.27 ± 4.63) cps
	6 mm	(162.06 ± 1.81) cps		6 mm	

4.2. Energy and Intensity of Secondary Scattering γ-rays from target

During the experiment, the $LaBr_3(Ce)$ detector array was fixed in its position, and secondary scattering γ-rays from the Ta target were measured to record their angular distribution. A typical set of background-subtracted energy spectra is shown in Fig. 11, measured at point R0.

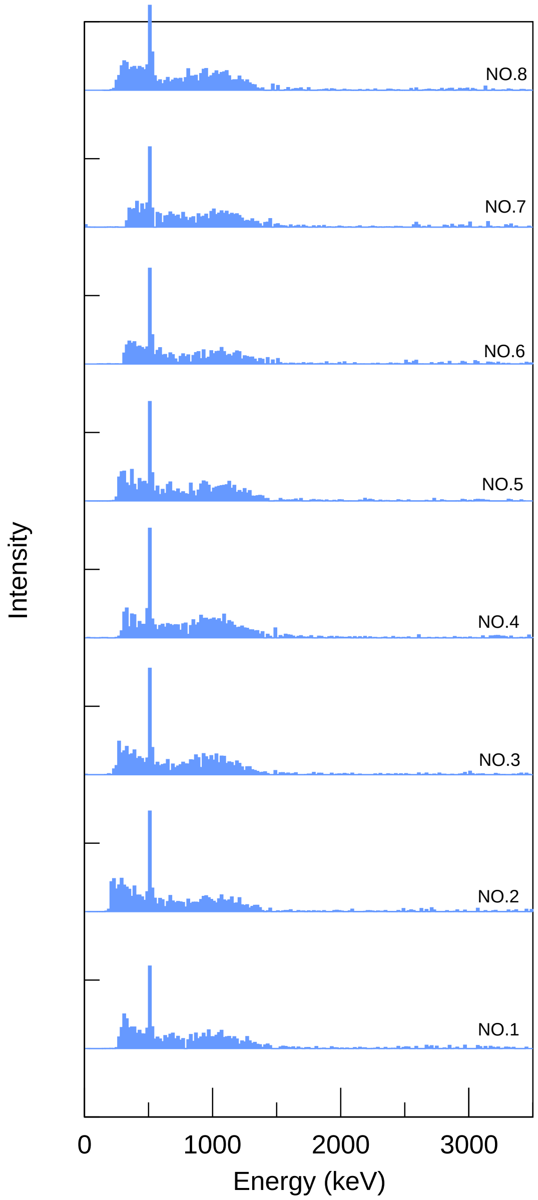

FIG. 11. The energy spectra of the secondary scattering γ-rays from the Ta target, measured by the $LaBr_3(Ce)$ detectors at R0. All backgrounds are subtracted.

5. DATA ANALYSIS

5.1. Processing of experimental data

Here, we take R0 as an example to present the processing flow and details. The azimuthal distribution of the raw data is shown in top left panel of Fig. 12. Experimental data processing comprises three sequential steps:

1. Background subtraction: bremsstrahlung, background of the $LaBr_3$(Ce) detectors and natural background were subtracted via laser-gated signal isolation. Raw spectra were partitioned into ON (laser-triggered) and OFF (background) components. Secondary scattering signals were extracted by subtracting OFF spectra from ON spectra, normalized by the laser duty cycle (time ratio = 1 in this study). The background-subtracted energy spectra are shown in Fig. 11. The resulting azimuthal distribution integrated over 550 keV to 1400 keV is shown in the top right panel of Fig. 12.

2. Detection efficiency correction: energy-dependent detector efficiency was convolved with background-subtracted spectra. The efficiency-corrected azimuthal distribution is shown in the bottom left panel of Fig. 12.

3. Geometric correction: solid-angle variations arising from fixed detector positions and synchronous target-beam motion were compensated using Monte Carlo-simulated correction factors. The final corrected azimuthal distribution is shown in the bottom right panel of Fig. 12.

5.2. Uncertainty

5.2.1. Statistical and background subtraction uncertainty

Uncertainties for all experimental data were calculated through error propagation, incorporating statistical errors, background subtraction uncertainties, efficiency errors, and geometric correction uncertainties. The statistical uncertainties follow Poisson statistics [19]. The raw spectra were partitioned into ON (laser-triggered) and OFF (background) spectra.

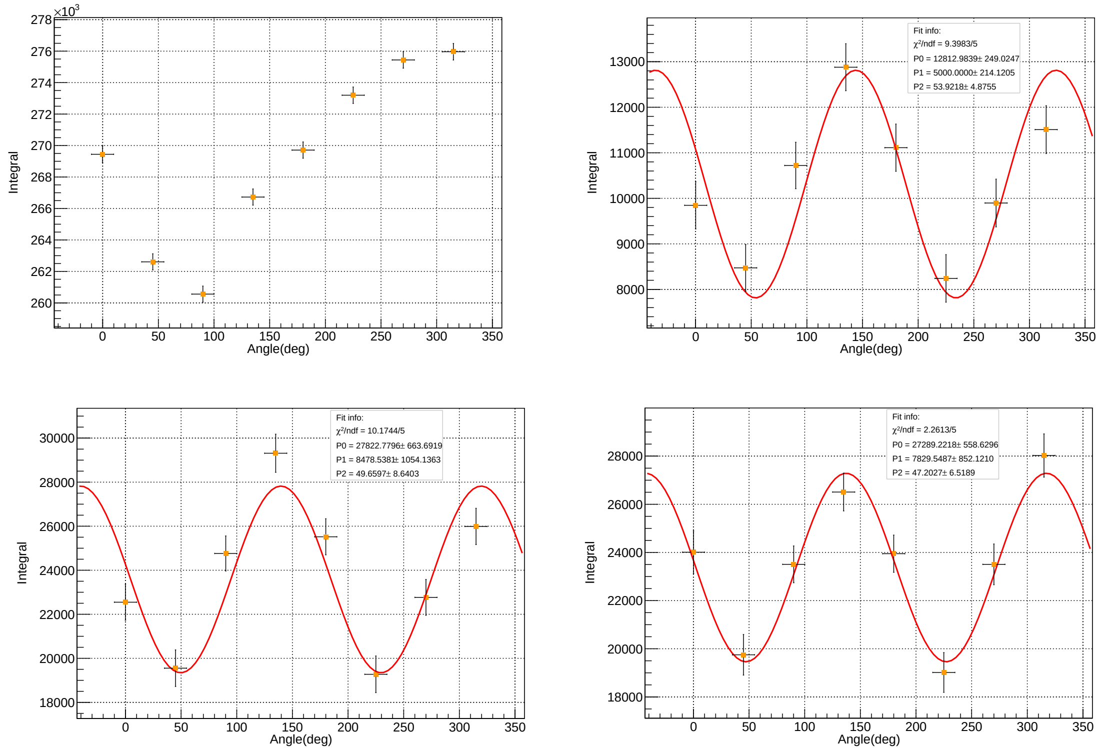

FIG. 12. The azimuthal distributions of the $LaBr_3(Ce)$ detectors array with raw data (in the top left panel), the background subtracted (in the top right panel), the corrected efficiency (in the bottom left panel) and the corrected geometry (in the bottom right panel), when the three-hole collimator is adjusted to R0. The red line represents the function fitting, and the fitting parameters are shown in the text box located in the blank area of the figure.

The combined statistical and background-subtraction uncertainty is given by

$$\Delta N_{\text{sub}} = \sqrt{{\Delta N_{\text{on}}}^2 + {\Delta N_{\text{off}}}^2} = \sqrt{N_{\text{on}} + N_{\text{off}}}. \tag{32}$$

where N_{on} and N_{off} denote counts in ON and OFF spectra, $\Delta N_{\text{on}} = \sqrt{N_{\text{on}}}$ and $\Delta N_{\text{off}} = \sqrt{N_{\text{off}}}$ represent statistical uncertainties, ΔN_{sub} is the net uncertainty after background subtraction.

5.2.2. Efficiency uncertainty

The intrinsic detection efficiency for all $LaBr_3(Ce)$ detectors with incident window area S exposed to a radioactive source of activity A is calculated as:

$$\eta_{\text{exp}} = \frac{N}{At\Omega} \tag{33}$$

where N is the measured count, t is the acquisition time, and $\Omega = \frac{S}{4\pi D^2}$ is the solid angle of the detector. This calibration was carried out at a distance D of 100 cm. The accuracy of the laser rangefinder is 5 mm, thus the solid angle uncertainty $\frac{\Delta\Omega}{\Omega} = |2\frac{\Delta D}{D}|$ is 1%. Here, the statistical uncertainties (much less than 1%) are negligible. The relative efficiency fluctuation σ_i are listed in column 8 and 10 of Table I. The total experimental efficiency uncertainty is:

$$\Delta\eta_{\mathrm{exp}} = \sqrt{\Delta\Omega^2 + {\sigma_i}^2} \tag{34}$$

Due to the limited calibration points, it is necessary to interpolate the efficiency curve simulated by Geant4 toolkit, where the step of energy is 10 keV, to obtain a continuous efficiency curve. A scaling coefficient f accounts for simulation-experiment discrepancies. Through least-squares minimization, we solve for f and its uncertainty. At reference energies 1173 keV and 1332 keV, the interpolated simulated efficiencies η_{sim1} and η_{sim2} correspond to experimental values η_{exp1} and η_{exp2}. The residual sum of squares is:

$$D = (f\eta_{\mathrm{sim1}} - \eta_{\mathrm{exp1}})^2 + (f\eta_{\mathrm{sim2}} - \eta_{\mathrm{exp2}})^2 \tag{35}$$

When D obtain a minimized value, the scale coefficient f is

$$f = \frac{\eta_{\mathrm{sim1}}\eta_{\mathrm{exp1}} + \eta_{\mathrm{sim2}}\eta_{\mathrm{exp2}}}{\eta_{\mathrm{sim1}}^2 + \eta_{\mathrm{sim2}}^2} = \frac{X}{Y} \tag{36}$$

According to the error propagation, the uncertainty of scaling coefficient Δf is derived as

$$\Delta f \approx \sqrt{\sum_{i=1}^{n} \left(\frac{\partial f}{\partial \eta_i}\Delta\eta_i\right)^2} \tag{37}$$

where $n = 4$, η_i is η_{sim1}, η_{sim2}, η_{exp1} and η_{exp2}, respectively. They are expanded as

$$\begin{cases} \dfrac{\partial f}{\partial \eta_{\mathrm{sim1}}}\Delta\eta_{\mathrm{sim1}} = \dfrac{\eta_{\mathrm{exp1}}Y - 2X\eta_{\mathrm{sim1}}}{Y^2}\Delta\eta_{\mathrm{sim1}} \\ \dfrac{\partial f}{\partial \eta_{\mathrm{sim2}}}\Delta\eta_{\mathrm{sim2}} = \dfrac{\eta_{\mathrm{exp2}}Y - 2X\eta_{\mathrm{sim2}}}{Y^2}\Delta\eta_{\mathrm{sim2}} \\ \dfrac{\partial f}{\partial \eta_{\mathrm{exp1}}}\Delta\eta_{\mathrm{exp1}} = \dfrac{\eta_{\mathrm{sim1}}}{Y}\Delta\eta_{\mathrm{exp1}} \\ \dfrac{\partial f}{\partial \eta_{\mathrm{exp2}}}\Delta\eta_{\mathrm{exp2}} = \dfrac{\eta_{\mathrm{sim2}}}{Y}\Delta\eta_{\mathrm{exp2}} \end{cases} \tag{38}$$

The final efficiency and its uncertainty are:

$$\eta = f\eta_{\mathrm{sim}}, \quad \Delta\eta = \sqrt{(f\Delta\eta_{\mathrm{sim}})^2 + (\eta_{\mathrm{sim}}\Delta f)^2} \tag{39}$$

where $\Delta\eta_{\mathrm{sim}}$ is the simulated statistical uncertainty.

5.2.3. Efficiency convolution

The detection efficiency curve $\eta(E)$ is convolved into the energy spectrum. The efficiency-corrected count integral from 550 keV to 1400 keV is

$$N_{\mathrm{con}} = \int_{550}^{1400} \frac{n(E)}{\eta(E)} \mathrm{d}E = \sum_{i(E=550)}^{i(E=1400)} \frac{n_i}{\eta_i} \tag{40}$$

where $n(E)$ is the measured energy spectrum; $\eta(E)$ is detection efficiency function; n_i and η_i are counts and efficiency in bin i, respectively. The uncertainty ΔN_{con} propagates as:

$$\Delta N_{\mathrm{con}} = \sqrt{\sum_{i(E=550)}^{i(E=1400)} (\frac{n_i}{\eta_i^2}\Delta\eta)^2 + \sum_{i(E=550)}^{i(E=1400)} (\frac{1}{\eta_i}\Delta n_i)^2} \tag{41}$$

5.2.4. Geometry correction uncertainty

The geometric correction coefficient f_Ω is the ratio of counts N_{act} in actual geometry to counts N_{sta} in ideal geometry

$$f_\Omega = \frac{N_{\mathrm{act}}}{N_{\mathrm{sta}}}, \quad \Delta f_\Omega = f_\Omega \sqrt{(\frac{\Delta N_{\mathrm{sta}}}{N_{\mathrm{sta}}})^2 + (\frac{N_{\mathrm{act}}}{N_{\mathrm{act}}})^2} \tag{42}$$

5.3. PVR and asymmetry

The PVR and asymmetry are calculated according to the definition and their uncertainties are obtained using the fitting parameters P_0 and P_1 and errors ΔP_0 and ΔP_1 according to the law of error propagation.

$$PVR = \frac{P_0}{P_0 - P_1}, \quad \Delta PVR = \sqrt{\left(\frac{P_1}{(P_0 - P_1)^2}\Delta P_0\right)^2 + \left(\frac{P_0}{(P_0 - P_1)^2}\Delta P_1\right)^2} \tag{43}$$

$$A_{\mathrm{exp}} = \frac{P_1}{2P_0 - P_1}, \quad \Delta A_{\mathrm{exp}} = \sqrt{\left(\frac{2P_1}{(2P_0 - P_1)^2}\Delta P_0\right)^2 + \left(\frac{2P_0}{(2P_0 - P_1)^2}\Delta P_1\right)^2} \tag{44}$$

The DOP and its uncertainty are given by

$$DOP = \frac{A_{\mathrm{exp}}}{A_{\mathrm{sim}}}, \quad \Delta DOP = \sqrt{\left(\frac{\Delta A_{\mathrm{exp}}}{A_{\mathrm{sim}}}\right)^2 + \left(\frac{A_{\mathrm{exp}}\Delta A_{\mathrm{sim}}}{A_{\mathrm{sim}}^2}\right)^2} \tag{45}$$

All fitting results, including extracted PVR, asymmetry A_{exp} and DOP are summarized in Table V. Several peripheral measurement points were excluded due to low γ-ray intensity and poor data quality away from the beam center.

TABLE V: Experimental Data Summary

Direction	R	Fit parameters				Experiment		DOP
		$\chi 2$	P_0	P_1	P_2	PVR	A_{exp}	
-	0 mm	2.26	$27\,289 \pm 559$	7832 ± 854	$(47 \pm 6)^\circ$	1.40 ± 0.63	0.17 ± 0.02	1.03 ± 0.13
0°	1 mm	0.39	$25\,218 \pm 606$	6643 ± 949	$(39 \pm 6)^\circ$	1.36 ± 0.07	0.15 ± 0.03	0.91 ± 0.18
	2 mm	11.40	$36\,125 \pm 1178$	$12\,253 \pm 1968$	$(61 \pm 4)^\circ$	1.51 ± 0.13	0.20 ± 0.04	1.16 ± 0.17
	3 mm	1.87	$35\,569 \pm 1168$	$12\,386 \pm 1896$	$(61 \pm 5)^\circ$	1.53 ± 0.13	0.21 ± 0.04	1.12 ± 0.17
	4 mm	9.92	$32\,949 \pm 1284$	$10\,284 \pm 2061$	$(75 \pm 6)^\circ$	1.45 ± 0.13	0.18 ± 0.04	0.88 ± 0.28
	5 mm	8.43	$46\,045 \pm 1969$	$18\,072 \pm 3013$	$(75 \pm 6)^\circ$	1.65 ± 0.18	0.24 ± 0.05	1.03 ± 0.21
	6 mm	31.80	$49\,204 \pm 1886$	$14\,769 \pm 2950$	$(95 \pm 9)^\circ$	1.43 ± 0.12	0.18 ± 0.04	0.68 ± 0.35
45°	1 mm	13.31	$30\,307 \pm 783$	7596 ± 1301	$(38 \pm 6)^\circ$	1.33 ± 0.08	0.14 ± 0.03	0.88 ± 0.23
	2 mm	7.36	$34\,713 \pm 807$	7346 ± 1303	$(35 \pm 6)^\circ$	1.27 ± 0.06	0.12 ± 0.02	0.67 ± 0.30
	3 mm	5.77	$38\,085 \pm 1066$	7806 ± 1750	$(29 \pm 6)^\circ$	1.26 ± 0.07	0.11 ± 0.03	0.58 ± 0.43
	4 mm	23.17	$87\,718 \pm 3249$	$31\,805 \pm 5476$	$(17 \pm 3)^\circ$	1.57 ± 0.16	0.22 ± 0.05	1.03 ± 0.21
	5 mm							
	6 mm							
90°	1 mm	1.78	$25\,673 \pm 521$	4089 ± 815	$(43 \pm 8)^\circ$	1.19 ± 0.04	0.09 ± 0.02	0.50 ± 0.44
	2 mm	5.92	$24\,414 \pm 719$	6174 ± 1193	$(26 \pm 5)^\circ$	1.34 ± 0.09	0.14 ± 0.03	0.75 ± 0.30
	3 mm	15.17	$20\,797 \pm 874$	7233 ± 1452	$(22 \pm 4)^\circ$	1.53 ± 0.17	0.21 ± 0.05	0.94 ± 0.26
	4 mm	3.23	$26\,484 \pm 1053$	8863 ± 1719	$(19 \pm 6)^\circ$	1.50 ± 0.15	0.20 ± 0.05	0.78 ± 0.30
	5 mm	15.94	$21\,805 \pm 901$	7104 ± 1522	$(0 \pm 10)^\circ$	1.48 ± 0.16	0.19 ± 0.05	0.69 ± 0.38
	6 mm	3.31	$19\,373 \pm 1148$	4726 ± 1887	$(15 \pm 11)^\circ$	1.32 ± 0.17	0.14 ± 0.06	0.50 ± 0.92
135°	1 mm	47.16	$30\,351 \pm 1956$	$14\,969 \pm 3680$	$(26 \pm 2)^\circ$	1.97 ± 0.49	0.33 ± 0.11	1.90 ± 0.18
	2 mm	3.98	$24\,455 \pm 520$	5444 ± 843	$(46 \pm 7)^\circ$	1.29 ± 0.06	0.13 ± 0.02	0.64 ± 0.28
	3 mm	18.21	$30\,871 \pm 1010$	$11\,125 \pm 1764$	$(31 \pm 4)^\circ$	1.56 ± 0.14	0.22 ± 0.04	0.99 ± 0.20
	4 mm	9.49	$26\,273 \pm 824$	7923 ± 1303	$(52 \pm 7)^\circ$	1.43 ± 0.10	0.18 ± 0.04	0.71 ± 0.28
	5 mm	17.77	$24\,149 \pm 1012$	8127 ± 1667	$(55 \pm 7)^\circ$	1.51 ± 0.16	0.20 ± 0.05	0.73 ± 0.34
	6 mm	11.02	$22\,016 \pm 1004$	$11\,331 \pm 1611$	$(40 \pm 7)^\circ$	2.06 ± 0.33	0.35 ± 0.07	1.22 ± 0.17
180°	1 mm	1.68	$27\,652 \pm 561$	5102 ± 864	$(47 \pm 7)^\circ$	1.23 ± 0.05	0.10 ± 0.02	0.59 ± 0.32

Continued on next page

TABLE V – continued from previous page

Direction	R	Fit parameters				Experiment		DOP
		$\chi 2$	P_0	P_1	P_2	PVR	A_{exp}	
	2 mm	7.22	$29\,027 \pm 827$	8817 ± 1346	$(58 \pm 5)^\circ$	1.44 ± 0.10	0.18 ± 0.03	1.01 ± 0.18
	3 mm	11.14	$26\,883 \pm 673$	6807 ± 1129	$(49 \pm 8)^\circ$	1.34 ± 0.08	0.15 ± 0.03	0.73 ± 0.26
	4 mm	10.33	$27\,307 \pm 1150$	9359 ± 1928	$(67 \pm 5)^\circ$	1.52 ± 0.17	0.21 ± 0.05	0.93 ± 0.27
	5 mm	5.76	$28\,372 \pm 1164$	$12\,352 \pm 1808$	$(82 \pm 6)^\circ$	1.77 ± 0.21	0.28 ± 0.05	1.14 ± 0.17
	6 mm	9.19	$26\,801 \pm 1337$	$14\,626 \pm 2019$	$(95 \pm 6)^\circ$	2.20 ± 0.39	0.38 ± 0.08	1.40 ± 0.14
225°	1 mm	11.43	$24\,476 \pm 585$	5005 ± 931	$(52 \pm 7)^\circ$	1.26 ± 0.06	0.11 ± 0.02	0.70 ± 0.30
	2 mm	15.74	$31\,751 \pm 1027$	$10\,110 \pm 1746$	$(54 \pm 6)^\circ$	1.47 ± 0.12	0.19 ± 0.04	1.06 ± 0.20
	3 mm	12.19	$29\,331 \pm 1248$	7766 ± 1995	$(32 \pm 8)^\circ$	1.36 ± 0.13	0.15 ± 0.05	0.76 ± 0.39
	4 mm	5.85	$36\,499 \pm 1859$	7335 ± 2921	$(2 \pm 28)^\circ$	1.25 ± 0.13	0.11 ± 0.05	0.50 ± 0.89
	5 mm	16.40	$26\,263 \pm 2507$	$14\,403 \pm 4259$	$(0 \pm 2)^\circ$	2.21 ± 0.84	0.38 ± 0.16	1.51 ± 0.28
	6 mm							
270°	1 mm	9.88	$24\,920 \pm 650$	6780 ± 1049	$(37 \pm 6)^\circ$	1.37 ± 0.08	0.16 ± 0.03	0.95 ± 0.19
	2 mm	2.34	$24\,721 \pm 648$	6689 ± 1045	$(35 \pm 6)^\circ$	1.37 ± 0.08	0.16 ± 0.03	0.85 ± 0.22
	3 mm	7.19	$27\,334 \pm 1100$	$11\,523 \pm 1804$	$(16 \pm 5)^\circ$	1.73 ± 0.20	0.27 ± 0.05	1.26 ± 0.16
	4 mm	6.15	$24\,292 \pm 1069$	9288 ± 1775	$(29 \pm 5)^\circ$	1.62 ± 0.20	0.24 ± 0.06	0.96 ± 0.25
	5 mm	12.71	$20\,877 \pm 1029$	6966 ± 1553	$(0 \pm 6)^\circ$	1.50 ± 0.17	0.20 ± 0.05	0.72 ± 0.38
	6 mm	4.13	$25\,317 \pm 1334$	2781 ± 2211	$(0 \pm 6)^\circ$	1.12 ± 0.11	0.06 ± 0.05	0.20 ± 4.14
315°	1 mm	6.30	$25\,748 \pm 535$	7237 ± 801	$(48 \pm 6)^\circ$	1.39 ± 0.06	0.16 ± 0.02	0.99 ± 0.13
	2 mm	2.45	$30\,158 \pm 611$	8661 ± 934	$(46 \pm 6)^\circ$	1.40 ± 0.06	0.17 ± 0.02	0.87 ± 0.15
	3 mm	9.83	$35\,289 \pm 1128$	$12\,775 \pm 1823$	$(59 \pm 4)^\circ$	1.57 ± 0.13	0.22 ± 0.04	1.08 ± 0.17
	4 mm	9.53	$37\,278 \pm 1047$	$13\,988 \pm 1638$	$(38 \pm 6)^\circ$	1.60 ± 0.12	0.23 ± 0.03	0.97 ± 0.15
	5 mm	6.97	$31\,829 \pm 1078$	$11\,273 \pm 1706$	$(52 \pm 7)^\circ$	1.55 ± 0.13	0.22 ± 0.04	0.79 ± 0.24

[1] Z. R. Hao, G. T. Fan, H. H. Xu, *et al.*, Quasi-monochromatic gamma beam modulation at SLEGS, Radiation Measurements **174**, 107127 (2024).

[2] L. X. Liu, H. Utsunomiya, G. T. Fan, *et al.*, Energy profile of laser Compton slant-scattering γ-ray beams determined by direct unfolding of total-energy responses of a BGO detector, Nuclear Instruments and Methods in Physics Research Section A: Accelerators, Spectrometers, Detectors and Associated Equipment **1063**, 169134 (2024).

[3] Y. X. Yang, Y. Zhang, Z. C. Li, *et al.*, Beam flux measurement using a photon activation analysis method at the SLEGS, Nuclear Science and Techniques **36**, 80 (2025).

[4] H. H. Xu, H. Utsunomiya, G. T. Fan, *et al.*, Gamma-ray flux in gated CW operation of CO_2 laser at SLEGS, Nuclear Instruments and Methods in Physics Research Section A: Accelerators, Spectrometers, Detectors and Associated Equipment **1073**, 170249 (2025).

[5] H. W. Wang, G. T. Fan, L. X. Liu, *et al.*, Commissioning of laser electron gamma beamline SLEGS at SSRF, Nuclear Science and Techniques **33**, 87 (2022).

[6] L. X. Liu, H. W. Wang, G. T. Fan, *et al.*, The SLEGS beamline of SSRF, Nuclear Science and Techniques **35**, 111 (2024).

[7] H. H. Xu, G. T. Fan, H. W. Wang, *et al.*, Interaction chamber for laser compton slant-scattering in SLEGS beamline at Shanghai Light Source, Nuclear Instruments and Methods in Physics Research Section a-Accelerators Spectrometers Detectors and Associated Equipment **1033**, 166742 (2022).

[8] Z. R. Hao, G. T. Fan, H. W. Wang, *et al.*, Collimator system of SLEGS beamline at Shanghai Light Source, Nuclear Instruments and Methods in Physics Research Section A -Accelerators Spectrometers Detectors and Associated Equipment **1013**, 165638 (2021).

[9] Z. R. Hao, G. T. Fan, H. W. Wang, *et al.*, A new annular collimator system of SLEGS beamline at Shanghai Light Source, Nuclear Instruments and Methods in Physics Research Section B-Beam Interactions with Materials and Atoms **519**, 9 (2022).

[10] K. J. Chen, L. X. Liu, Z. R. Hao, *et al.*, Simulation and test of the SLEGS TOF spectrometer at SSRF, Nuclear Science and Techniques **34**, 47 (2023).

[11] Z. R. Hao, H. H. Xu, G. T. Fan, *et al.*, Gamma spot monitor at SLEGS beamline, Nuclear Instruments and Methods in Physics Research Section A: Accelerators, Spectrometers, Detectors

and Associated Equipment **1068**, 169748 (2024).

[12] FARO, Quantum x faroarm® series (2025), accessed: 2025-04-15.

[13] FARO, Faro® cam2® software (2025), accessed: 2025-04-15.

[14] D. H. Goldstein, *Polarized Light*, 3rd ed. (CRC Press, 2011) p. 808.

[15] B. Schaefer, E. Collett, R. Smyth, D. Barrett, and B. Fraher, Measuring the stokes polarization parameters, American Journal of Physics **75**, 163 (2007).

[16] W. Greiner and J. Reinhardt, *Quantum Electrodynamics*, 4th ed. (2008).

[17] C. Sun and Y. K. Wu, Theoretical and simulation studies of characteristics of a Compton light source, Physical Review Special Topics - Accelerators and Beams **14**, 044701 (2011).

[18] S. Agostinelli, J. Allison, K. Amako, *et al.*, GEANT4-a simulation toolkit, Nuclear Instruments and Methods in Physics Research Section a-Accelerators Spectrometers Detectors and Associated Equipment **506**, 250 (2003).

[19] G. F. Knoll, *Radiation Detection and Measurement*, 4th ed. (John Wiley, Hoboken, N.J, 2010).
